# Clinical Profile, Epidemiology, and Outcomes of Granulomatous Amebic Encephalitis: A Systematic Review

**DOI:** 10.1093/ofid/ofag289

**Published:** 2026-05-12

**Authors:** Nitin Gupta, Shreya Singh, Kavita Salian, Sonali Singh, Shreya Das Adhikari, Mukund Gupta, Carl Boodman, Martin P Grobusch, Tirlangi Praveen Kumar

**Affiliations:** Department of Infectious Disease, Kasturba Medical College, Manipal Academy of Higher Education, Manipal, India; Department of Parasitology, Post Graduate Institute of Medical Education and Research, Chandigarh, India; Department of Infectious Disease, Kasturba Medical College, Manipal Academy of Higher Education, Manipal, India; Division of Neurology, Department of Pediatrics, Hospital for Sick Children, University of Toronto, Toronto, ON, Canada; Department of Anaesthesiology, Kasturba Medical College, Manipal Academy of Higher Education, Manipal, India; All India Institute of Medical Sciences, Jodhpur, Rajasthan, India; Department of Clinical Sciences, Institute of Tropical Medicine, Antwerp, Belgium; Center of Tropical Medicine and Travel Medicine, Department of Infectious Diseases, Amsterdam University Medical Centers, University of Amsterdam, Amsterdam Public Health – Global Health, Amsterdam Infection & Immunity, Amsterdam, The Netherlands; Masanga Medical Research Unit (MMRU), Masanga, Sierra Leone; Centre de Recherches Médicales en Lambaréné, Lambaréné, Gabon; Institut für Tropenmedizin und Deutsches Zentrum für Infektiologie (DZIF), Universität Tübingen, Tübingen, Germany; Institute of Molecular Medicine and Infectious Diseases, University of Cape Town, Cape Town, South Africa; Department of Infectious Disease, Kasturba Medical College, Manipal Academy of Higher Education, Manipal, India

**Keywords:** *Acanthamoeba*, amoeba, *Balamuthia*, GAE, granulomatous amoebic encephalitis

## Abstract

**Background:**

Granulomatous amebic encephalitis (GAE) is a rare but frequently fatal infection of the central nervous system, with poorly defined epidemiology, clinicoradiological features, and prognostic factors.

**Methods:**

We conducted a PRISMA-compliant systematic review of confirmed GAE cases registered with PROSPERO (CRD420251120627). MEDLINE, Embase, and Web of Science were searched from inception to September 2025. Two reviewers independently screened studies, extracted data, and assessed quality using the Joanna Briggs Institute checklist. Comparative analyses were performed between *Acanthamoeba* and *Balamuthia* infections and between survivors and nonsurvivors.

**Results:**

A total of 142 studies comprising 201 confirmed GAE cases were included. Cases were reported worldwide, predominantly from North America and Asia. Immunosuppression was documented in 58/196 (29.6%) cases. Headache, altered mental status, fever, and seizures were the most common presenting features. Neuroimaging most frequently demonstrated space-occupying lesions (148/190, 77.9%). *Balamuthia mandrillaris* accounted for 126/201 (62.7%) infections and *Acanthamoeba* for 67/201 (33.3%). Compared with *Balamuthia* infection, *Acanthamoeba* GAE was more often associated with immunocompromising conditions and meningeal involvement. Overall mortality was 146/191 (76.4%). Deaths were significantly more frequent in *Balamuthia* infection, and hypoglycorrhachia (50/101, 49.5%) emerged as a strong correlate of nonsurvival. Several antimicrobial agents were used more frequently among survivors, likely reflecting treatment intensity and survivor bias rather than definitive therapeutic efficacy.

**Conclusions:**

Granulomatous amebic encephalitis remains a devastating infection with persistently high mortality. Distinct organism-specific phenotypes and markers of disease severity may aid early diagnosis and prognostication.

## Teaser

Granulomatous amebic encephalitis remains a rare but highly fatal infection. This review highlights distinct phenotypes: *Acanthamoeba*, associated with immunosuppression and meningeal involvement, and *Balamuthia*, which predominates in immunocompetent hosts and is characterized by mass-like lesions and higher mortality, underscoring the importance of early organism-directed recognition.

Free-living ameba are associated with a spectrum of human disease ranging from localized infections such as keratitis and cutaneous lesions to pulmonary involvement and, in the most severe form, invasive central nervous system disease [1, 2]. Central nervous system disease due to free-living amebae is broadly classified into 2 major syndromes: primary amebic meningoencephalitis (PAM) and granulomatous amebic encephalitis (GAE). Primary amebic meningoencephalitis is a rapidly progressive, fulminant meningoencephalitis classically caused by *Naegleria fowleri*, typically affecting previously healthy individuals after freshwater exposure and characterized by an acute presentation with severe neutrophilic inflammation [1, 2]. In contrast, GAE usually has a more subacute-to-chronic presentation and is most commonly caused by *Acanthamoeba* spp. and *Balamuthia mandrillaris*. However, other free-living amebae have occasionally been implicated. In GAE, the pathogens invade the brain parenchyma and cerebral vasculature, leading to granulomatous inflammation, necrotizing vasculitis, and progressive tissue destruction [1, 2].

Human infection is thought to occur through inhalation of cysts or trophozoites via the respiratory tract or through direct inoculation via disrupted skin, followed by hematogenous dissemination to the brain [2, 3]. A definitive diagnosis relies on the direct demonstration of the organism in brain tissue or cerebrospinal fluid (CSF) [2, 4]. There is no established standard therapy for GAE, and outcomes remain poor despite the use of aggressive multidrug regimens [2, 5].

Previous reviews have been limited by small sample sizes, the inclusion of unconfirmed cases, or a focus on single organisms or specific geographic regions [3, 6, 7]. Moreover, comparative analyses between *Acanthamoeba* and *Balamuthia* infections, as well as systematic evaluation of factors associated with mortality, have been inconsistently performed [8]. Given the rarity of GAE, synthesizing individual patient-level data from microbiologically or histopathologically confirmed cases represents the most informative approach to delineating epidemiology, clinical phenotypes, diagnostic pathways, treatment practices, and outcomes. Accordingly, this systematic review aimed to comprehensively characterize confirmed GAE and to compare clinicoradiological features and mortality between *Acanthamoeba* and *Balamuthia* infections using individual patient-level data.

## METHODOLOGY

This systematic review was conducted and reported in accordance with the Preferred Reporting Items for Systematic Reviews and Meta-Analyses (PRISMA) guidelines (PRISMA Checklist in Supplementary File). The review protocol was prospectively registered with PROSPERO (CRD420251120627). A comprehensive literature search was conducted in MEDLINE (via PubMed), Embase, and Web of Science, spanning from database inception to September 2025. The search strategy combined controlled vocabulary (MeSH and Emtree terms) and free-text keywords related to free-living amebae (including *Acanthamoeba*, *Balamuthia*, and *Sappinia*), central nervous system infections (such as encephalitis and meningoencephalitis), and clinical outcomes (See the Search strategy in the Supplementary file). No language restrictions were applied, though the search was conducted in English. Reference lists of included articles and relevant reviews were hand-searched to identify additional eligible studies.

Studies were eligible if they reported individual patient data for confirmed GAE, defined by at least one of the following criteria: visualization of cysts or trophozoites on histopathology of brain tissue; positive molecular tests for a pathogenic free-living ameba from CSF or brain tissue; or positive microscopy or culture from CSF or brain tissue. Reports limited to keratitis or cutaneous/pulmonary disease without central nervous system involvement, as well as suspected or unconfirmed cases lacking microbiological or histopathological confirmation, were excluded. Cases due to *Naegleria fowleri* were excluded because this organism causes PAM, a distinct fulminant CNS syndrome that differs substantially from GAE in pathogenesis, clinical course, and diagnostic profile. Animal or in vitro studies, conference abstracts, and review articles without individual-level clinical data were excluded.

After duplicate removal, titles and abstracts were independently screened by 2 reviewers (K. S. and M. G.) using predefined eligibility criteria. Full texts of potentially relevant articles were then assessed for inclusion. Disagreements were resolved through discussion, with adjudication by a third reviewer (N. G.) when required. Data extraction was performed independently by 2 reviewers (K. S. and M. G.) using a standardized, piloted data collection form. Extracted variables included study characteristics (author, year, country), patient demographics (age, sex), comorbidities and risk exposures, presenting clinical features, neuroimaging findings, CSF parameters, diagnostic methods, identified amebic species, antimicrobial agents administered, reported treatment duration where available, and clinical outcome.

Comparative analyses were undertaken to evaluate differences in demographic characteristics, host risk factors, clinical presentation, CSF findings, neuroimaging features, diagnostic modalities, treatment patterns, and outcomes between *Acanthamoeba* and *Balamuthia* infections, as well as between survivors and nonsurvivors. Categorical variables were summarized as frequencies and percentages and compared using the chi-square test or Fisher's exact test, as appropriate, based on expected cell counts. Continuous variables were summarized as median with interquartile range and were compared using the Mann–Whitney *U* test. Analyses were conducted using available-case analysis, with denominators reported for each variable to reflect incomplete reporting across case reports. No imputation was performed for missing data. Given the case-based nature of the evidence, substantial heterogeneity in reporting, and the high likelihood of survivor and indication bias, particularly with respect to treatment variables, multivariable regression analyses were not performed. Temporal trends were assessed using Kendall's tau-b correlation, a nonparametric test for monotonic associations, given the non-normal distribution of the data. All analyses were considered exploratory and hypothesis-generating. A 2-sided *P*-value of <.05 was considered statistically significant.

Methodological quality was assessed independently by 2 reviewers using the Joanna Briggs Institute (JBI) Critical Appraisal Checklist for Case Reports [9]. Quality appraisal findings were summarized descriptively and used to contextualize the interpretation of results; however, they were not applied as exclusion criteria.

## RESULTS

### Screening, Eligibility, and Inclusion

**Figure 1.**
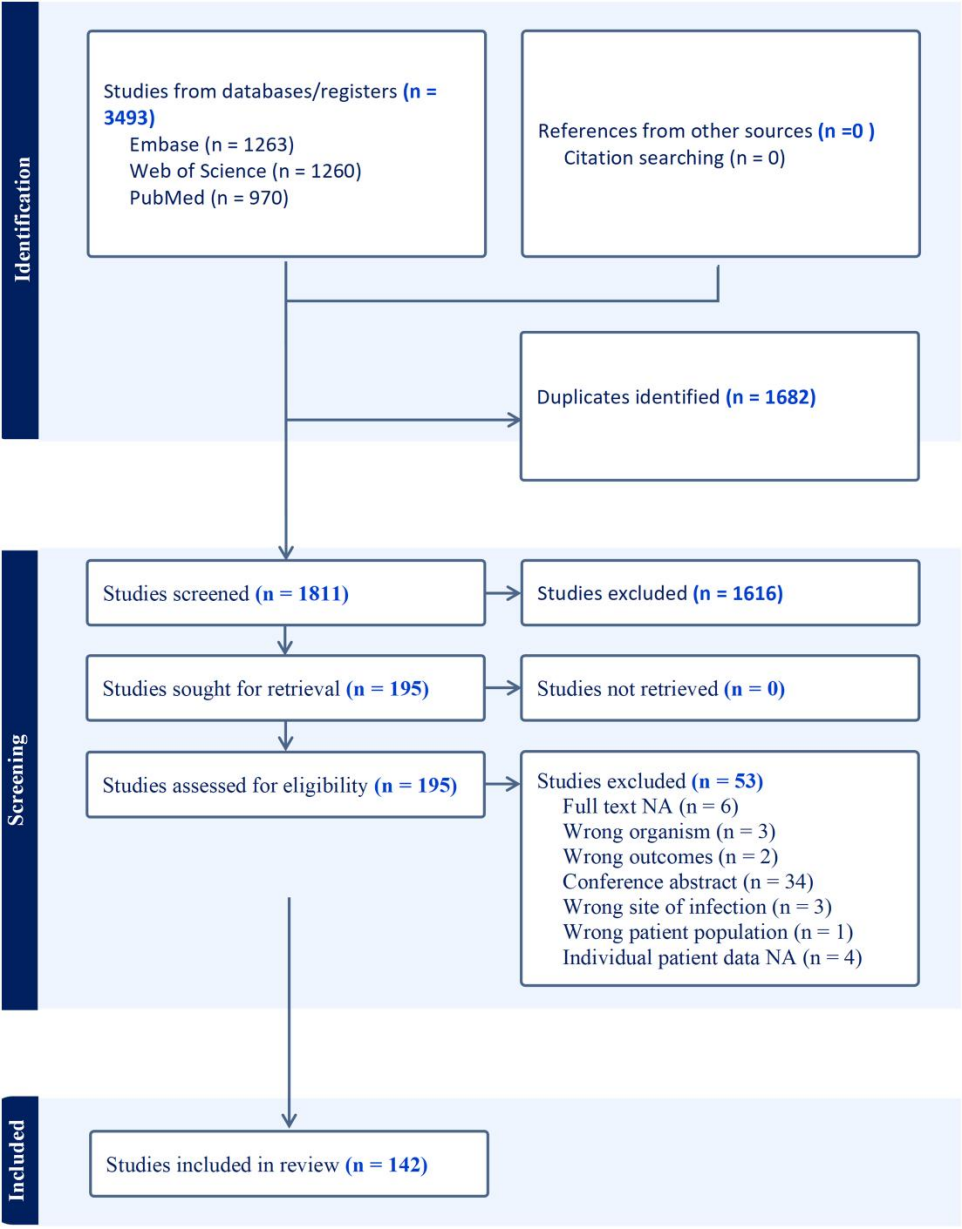
PRISMA flow diagram illustrating the identification, screening, eligibility assessment, and inclusion of studies reporting microbiologically or histopathologically confirmed cases of granulomatous amebic encephalitis.

The database search identified 3493 records across 3 major databases: Embase (n = 1263), Web of Science (n = 1260), and PubMed (n = 970) (Figure 1). After removal of 1682 duplicates, 1811 titles and abstracts were screened. Of these, 195 full-text articles were assessed, and 53 were excluded. A total of 142 studies were included, contributing individual-level data for 201 confirmed cases of GAE (Supplementary Table 1 with references).

### Baseline Demographic Characteristics

**Figure 2.**
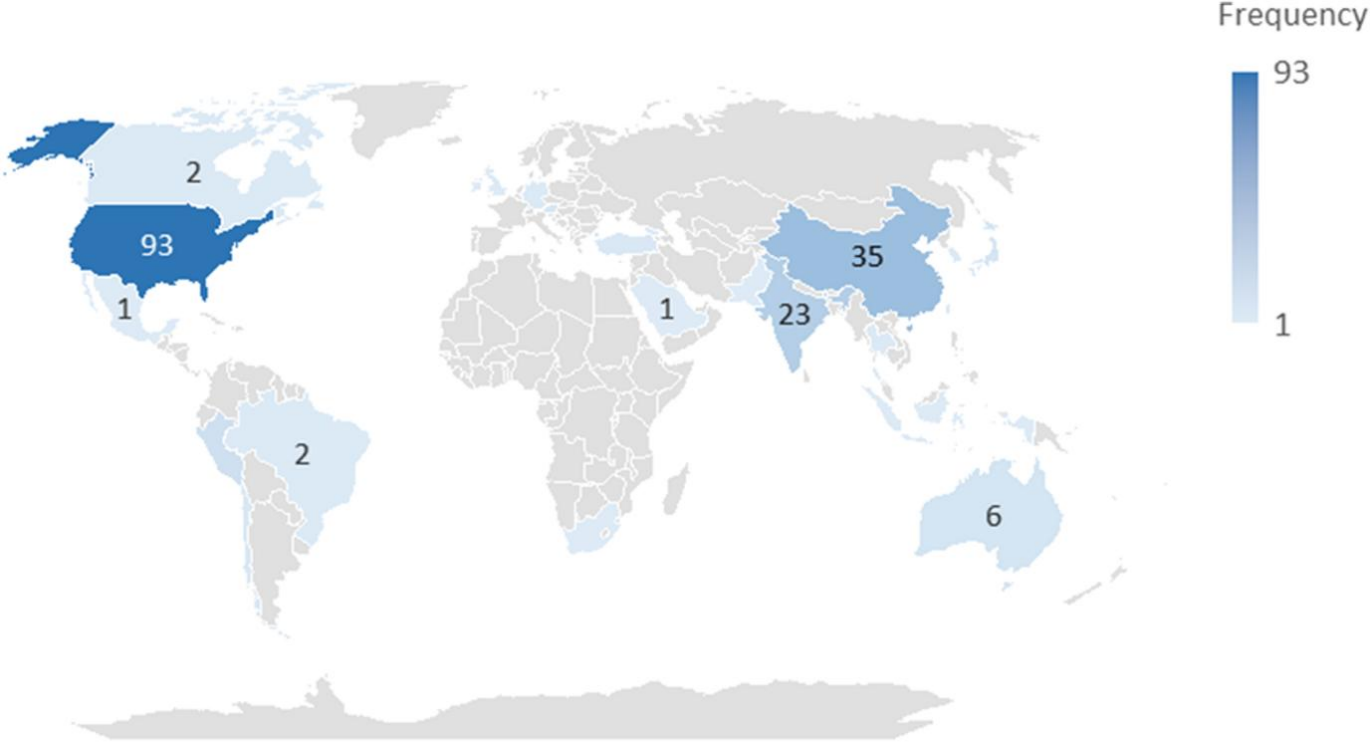
Global distribution of granulomatous amebic encephalitis (GAE) cases and etiologic agents. World map depicting the country-wise distribution of 201 confirmed GAE cases included in this systematic review. Shading intensity reflects the number of reported cases per country, with darker shades indicating higher frequencies, and numeric labels denoting total counts.

Cases were geographically widespread, with the majority reported from North America and Asia. The United States accounted for 93 cases (93/201, 46.3%), followed by China (35/201, 17.4%) and India (23/201, 11.4%) (Figure 2). Of the 67 *Acanthamoeba* cases with country-level data, most were from India (21/67, 31.3%) and the United States (22/67, 32.8%). Of the 126 cases of *Balamuthia mandrillaris,* most cases were from the United States (69/126, 54.8%) and China (34/126, 27.0%). Reports spanned from 1979 to 2025, with an increasing frequency over time. A Kendall's tau-b correlation (τ = 0.503, *P* < .001) demonstrated a significant positive monotonic trend between year and frequency, indicating a sustained increase in reported cases.

The median age was 31 (8–58) years, and 137/201 (68.2%) were male. A total of 74/201 (36.8%) patients were aged less than 18 years. Immunosuppression was documented in 58/196 (29.6%) patients and included diabetes mellitus, HIV infection, solid-organ or stem-cell transplantation, immunosuppressive drug exposure, and malignancy. Among the 14/196 (7.1%) patients with diabetes mellitus, only 1 was specifically described as having uncontrolled diabetes mellitus, whereas the degree of glycemic control was not adequately reported in the remaining cases. Specific immunosuppressive regimens were variably reported and included corticosteroids, cytotoxic chemotherapeutic agents, antimetabolites, and monoclonal antibodies. As many patients were receiving multiple agents concurrently and regimen details were often incomplete, corticosteroid exposure was extracted separately; among the 42/196 (21.4%) patients with reported immunosuppressant use, 29/42 (69.0%) had received corticosteroids, with variable doses and durations. Among the 14/196 (7.1%) transplanted patients, prior trimethoprim-sulfamethoxazole prophylaxis after transplantation and before GAE diagnosis was documented in 2/14 (14.3%). Environmental exposure to fresh water or soil was reported in 44/196 (22.4%), trauma in 22/196 (11.2%), and nasal irrigation in 4/196 (2.0%).

### Clinical Profile and Diagnosis

The duration of illness before diagnosis, reported in 110 patients, had a median of 14 days (interquartile range 7–30 days). Clinical features were reported in 194 patients. Headache occurred in 106/194 (54.6%), altered mental status in 95/194 (49.0%), fever in 85/194 (43.8%), seizures in 75/194 (38.7%), and motor weakness in 61/194 (31.4%). Cranial nerve palsies were documented in 49/194 (25.3%), and neck stiffness in 24/194 (12.4%). Cutaneous lesions, as evidenced by the presence of organisms in skin biopsy, were reported in 31/195 (15.9%) patients, with the face being the most commonly affected area (17 cases), followed by the trunk (5 cases), upper limbs (5 cases), and lower limbs (4 cases).

Mycobacterial coinfection was documented in 6 of 196 cases (3.1%). Among these, 2 patients were receiving immunosuppressive agents at the time of GAE diagnosis, whereas the remaining 4 were apparently immunocompetent. The coinfections included tuberculous meningitis in 4 patients, nontuberculous mycobacterial skin infection in 1 patient, and cutaneous leprosy in 1 patient.

Computed tomography (CT) scans were performed in 93 cases, and magnetic resonance imaging (MRI) scans in 170. Among patients with interpretable neuroimaging findings, space-occupying or ring-enhancing lesions were most common (148/190, 77.9%), followed by leptomeningeal enhancement (57/190, 30.0%) and hydrocephalus (25/190, 13.2%), while intracranial hemorrhage was observed in 24/189 (12.7%). Basal exudates were infrequently reported (3/190, 1.6%).

CSF analysis demonstrated pleocytosis in 115/122 (94.3%) and elevated protein in 104/120 (86.7%). Among cases with a reported CSF cellular differential (n = 83), lymphocytic pleocytosis was observed in 66 (79.5%) patients. Among the 101 patients with interpretable CSF glucose values, hypoglycorrhachia was present in 50/101 (49.5%).

Diagnosis was established by microscopy in 183/189 (96.8%), molecular testing in 137/140 (97.9%), and culture in 31/45 (68.9%). Molecular identification methods were heterogeneous and included genus- or species-specific PCR, broad-range PCR with sequencing confirmation, and other molecular approaches, as reported. Culture techniques were not standardized across reports. *Balamuthia mandrillaris* was identified in 126/201 (62.7%) of cases, and *Acanthamoeba* in 67/201 (33.3%). Of the remaining cases, *Vermamoeba vermiformis* was identified in 2 patients, *Sappinia diploidea* in one patient, and no genus was specified in 5 cases [10, 11]. Genotypic characterization was reported in 10 cases, with genotype T4 identified in 5 cases, followed by T1 (2 cases) and T5, T12, and T18 (1 case each).

A brain biopsy report was available in 104 of 201 patients (51.7%). Diagnostic tissue was obtained ante-mortem in 74 patients (74/201, 36.8%) and postmortem in 39 patients (39/201, 19.4%), with 9 patients undergoing both ante-mortem and postmortem biopsy. Among biopsied patients (n = 104), vasculitic changes, defined by inflammatory or necrotizing involvement of vessel walls with preferential perivascular localization of amebic organisms, were identified in 60 cases (57.7%). Parenchymal necrosis was documented in 58 cases (55.8%), while granulomatous inflammation was observed in 36 cases (34.6%). Among the 70 patients in whom amebic morphology was explicitly described on histopathology, trophozoites were identified in 65 cases (92.9%) and cyst forms in 33 cases (47.1%), frequently coexisting within the same specimen and often in close association with areas of necrosis and vasculitis. Among patients with reported macroscopic descriptions of biopsy or autopsy specimens (n = 61), gross intracranial hemorrhage was observed in 26 cases (42.6%), consistent with the angiocentric and hemorrhagic pathological phenotype of granulomatous amebic encephalitis.

### Treatment and Outcomes

Treatment data were available for 166 patients. For analysis, active antiamebic therapy was defined as treatment regimens that included one or more agents with reported activity against free-living amebae in published literature, including miltefosine, metronidazole, fluconazole, flucytosine, amphotericin B, azithromycin, rifampicin, trimethoprim–sulfamethoxazole, and pentamidine. Overall, 127/166 patients (76.5%) with available treatment data received at least one predefined antiamebic agent. Among the 127 patients who received at least one predefined antiamebic agent, fluconazole was the most frequently used drug (84/127, 66.1%), followed by azithromycin (50/127, 39.4%), trimethoprim–sulfamethoxazole (47/127, 37.0%), flucytosine (42/127, 33.1%), miltefosine (41/127, 32.3%), amphotericin B (40/127, 31.5%), and rifampicin (38/127, 29.9%). Pentamidine was used in 35/127 (27.6%), while metronidazole was administered in 24/127 (18.9%). Of these 127 patients, 103 (81.1%) received 2 or more drugs in combination. Treatment duration was reported in 101 patients, with a median duration of 30 days (IQR: 8.5–123 days). Although overall treatment duration was reported, distinctions between therapy phases and the use of secondary prophylaxis were inconsistently described. Outcome data were available for 191 patients, among whom mortality remained high at 146/191 (76.4%).

### Comparison of *Acanthamoeba* vs *Balamuthia*

Patients with *Acanthamoeba* GAE were significantly older than those with *Balamuthia* infection (median age 33 years [IQR 18.5–63] vs 25 years [IQR 7–56.5], *P* = .032). They were more likely to have underlying immunosuppression (34/67 [50.7%] vs 21/121 [17.4%], *P* < .001) (Table 1). Clinically, headache was more frequent in *Balamuthia* GAE (71/120 [59.2%] vs 29/66 [43.9%], *P* = .046), whereas neck stiffness was more commonly observed in *Acanthamoeba* infection (13/66 [19.7%] vs 11/120 [9.2%], *P* = .040). Neuroimaging demonstrated marked differences between the 2 etiologies: space-occupying or ring-enhancing lesions predominated in *Balamuthia* GAE (104/120 [86.7%] vs 38/62 [61.3%], *P* < .001), while leptomeningeal enhancement (27/62 [43.5%] vs 27/120 [22.5%], *P* = .003) and basal exudates (3/62 [4.8%] vs 0/120 [0%], *P* = .015) were more frequently associated with *Acanthamoeba* infection. Although CSF pleocytosis was common in both groups, hypoglycorrhachia occurred significantly more often in *Balamuthia* infection (34/59 [57.6%] vs 13/39 [33.3%], *P* = .018). In addition, a lymphocytic CSF pattern (29/67 [43.3%] vs 35/126 [27.8%], *P* = .031) was more frequently observed in *Acanthamoeba* GAE, whereas histopathological evidence of vasculitis was more common in *Balamuthia* infection (43/71 [60.6%] vs 16/40 [40.0%], *P* = .037).

**Table 1.** Comparison of Clinical Characteristics, Diagnostics, Treatment, and Outcomes Between *Acanthamoeba* and *Balamuthia* Granulomatous Amebic Encephalitis

Variable	*Acanthamoeba* spp.	*Balamuthia* spp.	*P* Value
Male sex	48/67 (71.6%)	83/126 (65.9%)	.414
Median age (years)	33 (18.5–63)	25 (7–56.5)	.032
Immunosuppression	34/67 (50.7%)	21/121 (17.4%)	<.001
Environmental exposure	13/67 (19.4%)	29/121 (24.0%)	.472
Trauma history	5/67 (7.5%)	17/121 (14.0%)	.178
Duration of illness at presentation (days), median	15 (7–31.5)	14.5 (8.5–30)	.713
Fever	35/66 (53.0%)	47/120 (39.2%)	.068
Headache	29/66 (43.9%)	71/120 (59.2%)	.046
Altered sensorium	36/66 (54.5%)	53/120 (44.2%)	.175
Seizures	27/66 (40.9%)	46/120 (38.3%)	.731
Motor weakness	21/66 (31.8%)	38/120 (31.7%)	.983
Cranial nerve palsy	11/66 (16.7%)	35/120 (29.2%)	.059
Neck stiffness	13/66 (19.7%)	11/120 (9.2%)	.040
Skin involvement	7/66 (10.6%)	24/121 (19.8%)	.105
SOL/ring-enhancing lesion	38/62 (61.3%)	104/120 (86.7%)	<.001
Leptomeningeal enhancement	27/62 (43.5%)	27/120 (22.5%)	.003
Basal exudates	3/62 (4.8%)	0/120 (0%)	.015
Hydrocephalus	10/62 (16.1%)	15/120 (12.5%)	.500
Intracranial hemorrhage (HMG)	11/62 (17.7%)	12/119 (10.1%)	.142
CSF pleocytosis	44/47 (93.6%)	67/71 (94.4%)	.866
Elevated CSF protein	38/43 (88.4%)	61/72 (84.7%)	.584
Lymphocytic CSF pattern	29/67 (43.3%)	35/126 (27.8%)	.031
Gross hemorrhage on the specimen	7/22 (31.8%)	17/36 (47.2%)	.248
Hypoglycorrhachia	13/39 (33.3%)	34/59 (57.6%)	.018
Vasculitis on histopathology	16/41 (39.0%)	43/71 (60.6%)	.028
Biopsy necrosis	20/36 (55.6%)	36/63 (57.1%)	.878
Biopsy granuloma	10/36 (27.8%)	25/63 (39.7%)	.233
Trophozoite identified on biopsy	22/26 (84.6%)	38/39 (97.4%)	.057
Cyst identified on biopsy	17/26 (65.4%)	15/39 (38.5%)	.033

Abbreviations: CSF, cerebrospinal fluid; SOL, space-occupying lesion.

### Comparison of Immunosuppression vs no Immunosuppression

Compared with patients without immunosuppression, those with immunosuppression were more likely to have *Acanthamoeba* infection (34/58, 58.6% vs 33/138, 23.9%; *P* < .001), leptomeningeal enhancement (24/58, 41.4% vs 33/132, 25.0%; *P* = .023), intracranial hemorrhage (13/58, 22.4% vs 11/131, 8.4%; *P* = .008), and cyst forms on histopathology (18/27, 66.7% vs 15/43, 34.9%; *P* = .010), whereas biopsy granuloma (29/64, 45.3% vs 7/40, 17.5%; *P* = .004) and hypoglycorrhachia (41/71, 57.7% vs 9/30, 30.0%; *P* = .011) were more common among patients without immunosuppression (Table 2). Mortality did not differ significantly between the 2 groups (46/56, 82.1% vs 100/135, 74.1%; *P* = .232).

**Table 2.** Comparison of Clinical Characteristics, Diagnostics, Treatment, and Outcomes Between Patients With no Immunosuppression and Those With Immunosuppression

Variable	No Immunosuppression	Immunosuppression	*P* value
*Acanthamoeba* spp.	33/138 (23.9%)	34/58 (58.6%)	<.001
*Balamuthia* spp.	100/138 (72.5%)	21/58 (36.2%)	
Fever	58/137 (42.3%)	27/57 (47.4%)	.520
Headache	80/137 (58.4%)	26/57 (45.6%)	.103
Altered sensorium	62/137 (45.3%)	33/57 (57.9%)	.109
Seizures	49/137 (35.8%)	26/57 (45.6%)	.199
Motor weakness	42/137 (30.7%)	19/57 (33.3%)	.715
Cranial nerve palsy	40/137 (29.2%)	9/57 (15.8%)	.050
Neck stiffness	19/137 (13.9%)	5/57 (8.8%)	.326
Skin involvement	23/138 (16.7%)	8/57 (14.0%)	.648
SOL/ring-enhancing lesion	99/132 (75.0%)	49/58 (84.5%)	.147
Leptomeningeal enhancement	33/132 (25.0%)	24/58 (41.4%)	.023
Basal exudates	2/132 (1.5%)	1/58 (1.7%)	.915
Hydrocephalus	19/132 (14.4%)	6/58 (10.3%)	.447
Intracranial hemorrhage (HMG)	11/131 (8.4%)	13/58 (22.4%)	.008
CSF pleocytosis	78/82 (95.1%)	37/40 (92.5%)	.559
Elevated CSF protein	67/81 (82.7%)	37/39 (94.9%)	.067
Gross hemorrhage on the specimen	14/39 (35.9%)	12/22 (54.5%)	.157
Hypoglycorrhachia	41/71 (57.7%)	9/30 (30.0%)	.011
Vasculitis on histopathology	40/72 (55.6%)	20/46 (43.5%)	.201
Biopsy necrosis	37/64 (57.8%)	21/40 (52.5%)	.596
Biopsy granuloma	29/64 (45.3%)	7/40 (17.5%)	.004
Trophozoite identified on biopsy	41/43 (95.3%)	24/27 (88.9%)	.307
Cyst identified on biopsy	15/43 (34.9%)	18/27 (66.7%)	.010
Death	100/135 (74.1%)	46/56 (82.1%)	.232

Abbreviations: CSF, cerebrospinal fluid; SOL, space-occupying lesion.

### Factors Associated With Poor Outcomes

Outcomes differed significantly by etiologic agent, with *Acanthamoeba* infection occurring more frequently among survivors (20/45, 44.4%) compared with nonsurvivors (44/146, 30.1%), whereas *Balamuthia* infection predominated among nonsurvivors (100/146, 68.5% vs 25/45, 55.6% among survivors; *P* = .003) (Table 3). CSF hypoglycorrhachia was strongly associated with mortality, occurring in 43/76 nonsurvivors (56.6%) compared with 7/25 survivors (28.0%) (*P* = .013). Parenchymal necrosis on biopsy was more frequent among nonsurvivors than survivors (50/81, 61.7% vs 7/19, 36.8%; *P* = .049), suggesting more advanced or destructive disease at presentation. In addition, the presence of cyst forms on histopathology was significantly associated with mortality, identified in 29/58 nonsurvivors (50.0%) compared with 1/8 survivors (12.5%) (*P* = .046), consistent with recognized therapeutic resistance and the persistence of the cystic stage. Several antimicrobial agents were used significantly more frequently among survivors than nonsurvivors, including fluconazole (34/44, 77.3% vs 48/117, 41.0%; *P* < .001), trimethoprim–sulfamethoxazole (25/44, 56.8% vs 21/117, 17.9%; *P* < .001), rifampicin (16/44, 36.4% vs 21/117, 17.9%; *P* = .013), and flucytosine (16/44, 36.4% vs 24/117, 20.5%; *P* = .038). Among patients who received at least one antiamebic drug, 41/43 (95.3%) survivors received 2 or more drugs, compared with 58/80 (72.5%) nonsurvivors (*P* = .002). Inspection of individual treatment regimens revealed marked heterogeneity, with no single drug combination consistently associated with improved survival.

**Table 3.** Comparison of Clinical Characteristics, Diagnostics, and Treatment Between Survivors and Nonsurvivors With Granulomatous Amebic Encephalitis

Variable	Survivors (n = 45)	Nonsurvivors (n = 146)	*P* value
Male sex	33 (73.3%)	98 (67.1%)	.433
Immunosuppression	10 (22.2%)	46 (31.5%)	.232
Fever	19 (42.2%)	66 (45.5%)	.698
Headache	22 (48.9%)	81 (55.9%)	.412
Altered sensorium	21 (46.7%)	73 (50.3%)	.666
Seizures	23 (51.1%)	51 (35.2%)	.055
Cranial nerve palsy	12 (26.7%)	35 (24.1%)	.731
SOL/ring-enhancing lesion	30 (69.8%)	113 (79.6%)	.179
Hydrocephalus	3 (7.0%)	22 (15.5%)	.152
Leptomeningeal enhancement	13 (30.2%)	42 (29.6%)	.934
Basal exudates	1 (2.3%)	2 (1.4%)	.677
CSF pleocytosis	28 (87.5%)	87 (96.7%)	.055
Hypoglycorrhachia	7 (28.0%)	43 (56.6%)	.013
*Acanthamoeba* infection	20 (44.4%)	44 (30.1%)	—
*Balamuthia* infection	25 (55.6%)	100 (68.5%)	.003
Gross hemorrhage on the specimen	1/7 (14.3%)	24/52 (46.2%)	.109
Vasculitis on biopsy	12/24 (50.0%)	48/90 (53.3%)	.771
Biopsy necrosis	7/19 (36.8%)	50/81 (61.7%)	.049
Biopsy granuloma	7/19 (36.8%)	28/81 (34.6%)	.852
Trophozoite identified on biopsy	8/8 (100%)	54/58 (93.1%)	.443
Cyst identified on biopsy	1/8 (12.5%)	29/58 (50.0%)	.046
Miltefosine	14 (31.8%)	25 (21.4%)	.168
Metronidazole	7 (15.9%)	17 (14.5%)	.827
Fluconazole	34 (77.3%)	48 (41.0%)	<.001
Flucytosine	16 (36.4%)	24 (20.5%)	.038
Amphotericin B	9 (20.5%)	30 (25.6%)	.494
Azithromycin	14 (31.8%)	34 (29.1%)	.733
Rifampicin	16 (36.4%)	21 (17.9%)	.013
Trimethoprim–sulfamethoxazole	25 (56.8%)	21 (17.9%)	<.001
Pentamidine	11 (25.0%)	21 (17.9%)	.318

Abbreviations: CSF, cerebrospinal fluid; SOL, space-occupying lesion.

### Critical Appraisal Summary

Patient demographics were reported in all cases (201/201, 100%) (Supplementary Table 2). A clinical history with a clear timeline was available in 110 reports (54.7%), while presentation details were described in 194 reports (96.5%). Diagnostic confirmation was clearly reported in 196 cases (97.5%). Treatment details were available in only 103 reports (51.2%), representing the most common reporting limitation.

## DISCUSSION

In this systematic review encompassing 201 confirmed cases of GAE reported over more than 4 decades, we demonstrate that GAE remains a globally distributed and persistently devastating infection of the central nervous system. Despite advances in diagnostic techniques and the increasing use of combination antimicrobial regimens, mortality remained high at over 75%, underscoring the continued lethality of this condition. Importantly, a substantial proportion of cases, particularly those caused by *Balamuthia mandrillaris,* occur in individuals without recognized immunosuppression, emphasizing that GAE is not confined to classically immunocompromised hosts and may present unexpectedly in individuals who appear immunocompetent.

The apparent rarity and geographical clustering of reported GAE cases likely reflect substantial underdiagnosis and reporting bias rather than true geographic restriction, given the ubiquitous environmental presence of free-living amebae [3]. The observed increase in reported cases over time is likely multifactorial, reflecting improved diagnostic awareness and access to neuroimaging and molecular testing, alongside environmental and climatic changes that may increase freshwater exposure and opportunities for infection [12–15]. It is worth noting that the geographic patterns of keratitis and other extra-CNS manifestations may differ and were not evaluated in this study.

Most patients presented with a subacute illness, with a median symptom duration of 14 days before hospital presentation, reflecting the distinct pathogenesis of GAE, which differs fundamentally from that of primary amebic meningoencephalitis caused by *Naegleria fowleri* [8]. Within the central nervous system, *Acanthamoeba* and *Balamuthia* predominantly elicit a cell-mediated immune response characterized by macrophage and T-lymphocyte infiltration and granuloma formation, rather than the intense neutrophilic inflammation typical of *Naegleria* infection, which accounts for the more indolent clinical course of GAE [8]. Although GAE has traditionally been regarded as a disease of immunocompromised individuals, including those with HIV/AIDS, malignancy, uncontrolled diabetes mellitus, or exposure to immunosuppressive therapies [16–18], most patients in this review had no overt immunosuppression, underscoring the need for heightened clinical suspicion even in immunocompetent hosts presenting with subacute encephalitis or focal intracranial lesions.

Several clinically meaningful epidemiological and phenotypic patterns emerged. The predominance of headache and altered mental status likely reflects raised intracranial pressure and diffuse parenchymal involvement. At the same time, seizures and focal motor deficits are consistent with cortical irritation from focal lesions, in keeping with the high frequency of mass-like or ring-enhancing abnormalities on neuroimaging [19]. Cranial nerve palsies, although less common, suggest brainstem or meningeal involvement, whereas the relative infrequency of neck stiffness highlights the frequent absence of classical meningeal signs [14, 19–21]. Fever was uncommon, further compounding diagnostic uncertainty. Cutaneous lesions were reported in only a minority of cases. However, they may provide an important diagnostic clue and a readily accessible site for tissue sampling; their infrequent documentation suggests that skin involvement may be under-recognized or under-reported [21–23]. Given that cutaneous disease can precede neurological manifestations, careful dermatological examination should be an integral part of the evaluation of unexplained encephalitis.

Neuroimaging abnormalities were almost universal and most commonly manifested as ring-enhancing or space-occupying lesions [19, 24]. These findings frequently led to misdiagnosis as intracranial abscesses, tuberculomas, or neoplasms and often prompted empirical antimicrobial or antitubercular therapy, delaying definitive diagnosis [19]. Less frequent findings, including leptomeningeal enhancement and hydrocephalus, highlight that GAE exists along a pathological spectrum ranging from focal parenchymal disease to diffuse meningoencephalitic involvement [11, 14, 15, 20, 25].

Diagnostic approaches varied substantially across studies, reflecting both temporal evolution and resource availability [2, 26]. Microscopy remained the most frequently reported modality, particularly in earlier reports and resource-limited settings, where diagnosis often relied on brain biopsy or postmortem examination. In more recent cases, molecular techniques, most commonly genus-specific or broad-range PCR, have increasingly contributed to diagnosis, enabling species-level identification from small tissue samples or CSF. Despite these advances, diagnosis frequently required invasive sampling, underscoring the persistent difficulty of recognizing GAE early in its course. CSF findings were largely nonspecific; however, hypoglycorrhachia consistently emerged as a marker of poor outcome, likely reflecting advanced inflammatory disease, extensive parenchymal involvement, or impaired cerebral glucose metabolism [2, 27]. Similar associations have been reported in prior clinicopathological studies, where profound CSF abnormalities correlated with advanced disease and fatal outcomes [5].

Clear organism-specific differences were evident. Patients with *Balamuthia* infection were significantly younger and less likely to be immunosuppressed, reinforcing prior observations that this pathogen frequently affects apparently immunocompetent hosts and may therefore evade early clinical suspicion [8, 28]. In contrast, *Acanthamoeba* GAE occurred more often in immunosuppressed individuals, suggesting a greater role for host immune dysfunction. Distinct clinicoradiological phenotypes were also observed: *Balamuthia* GAE was characterized by a predominance of mass-like parenchymal lesions, whereas *Acanthamoeba* infection was more frequently associated with meningeal involvement, including leptomeningeal enhancement, basal exudates, and lymphocytic CSF patterns. Histopathological evidence of vasculitis was more common in *Balamuthia* infection, consistent with its recognized angioinvasive propensity. Together, these findings extend prior reports that *Acanthamoeba* tends to produce greater meningeal involvement, while *Balamuthia* more often manifests as a parenchymal mass-like disease [8, 28, 29].

The organisms' ability to exist in both trophozoite and cyst forms further complicates host clearance and therapeutic response, as cysts demonstrate marked resistance to antimicrobial agents and environmental stressors [3]. In our review, the presence of cystic forms on histopathology was associated with increased mortality and extensive parenchymal necrosis, which was also more frequent among nonsurvivors. Necrosis may further limit antimicrobial penetration into infected tissue, collectively contributing to poor therapeutic responses and adverse outcomes. Therapeutic strategies were highly heterogeneous, with most patients receiving empiric combination regimens incorporating azoles, miltefosine, antibacterial agents, or adjunctive therapies [30, 31]. No single agent or combination consistently emerged as superior, and apparent associations between specific drugs and survival were inconsistent and likely confounded by disease severity, treatment timing, and survivor bias [5]. Overall, the persistently high mortality despite aggressive multidrug therapy highlights the urgent need for earlier diagnosis, standardized treatment protocols, and novel therapeutic approaches. The possible role of secondary prophylaxis in selected high-risk patients remains uncertain. Although trimethoprim-sulfamethoxazole and azoles were included in several treatment regimens, the observational, highly heterogeneous nature of the available literature precludes any recommendation for routine prophylaxis.

The review has several limitations inherent to the available literature. First, a substantial proportion of patients were diagnosed only at postmortem examination, raising the possibility that overall mortality may be overestimated, as rapidly fatal or diagnostically challenging cases are more likely to be recognized and reported. Second, the analysis is primarily based on case reports and small case series, which are susceptible to reporting bias, incomplete data, and nonstandardized definitions. Third, missing data were common for several key variables, particularly treatment details and duration, limiting comparative analyses. Fourth, temporal changes in diagnostic modalities and treatment availability over the long study period may have influenced outcomes in ways that could not be fully accounted for. Fifth, associations between treatment and survival should not be interpreted as causal, given the high likelihood of survivor bias and confounding by disease severity. Finally, although this review followed a prespecified search period ending in September 2025, subsequently published rare cases, including those suggesting underlying inborn errors of immunity as a potential host susceptibility factor, were not captured in the present analysis [32].

## CONCLUSIONS

GAE remains a rare but overwhelmingly lethal central nervous system infection, with mortality exceeding 70% despite advances in diagnostics and multidrug therapy. Distinct epidemiological, clinical, and radiological differences between *Acanthamoeba* and *Balamuthia* infections may aid early diagnostic suspicion, but current treatment strategies remain empiric and inconsistently reported. Standardized reporting of therapeutic regimens, including duration, along with earlier molecular diagnosis and collaborative prospective data collection, are urgently needed to improve outcomes in this neglected and often fatal disease.

## Supplementary Material

ofag289_Supplementary_Data

## Supplementary Data

Supplementary materials are available at *Open Forum Infectious Diseases* online. Consisting of data provided by the authors to benefit the reader, the posted materials are not copyedited and are the sole responsibility of the authors, so questions or comments should be addressed to the corresponding author.

## References

[1] Kot K, Łanocha-Arendarczyk N, Kosik-Bogacka D. Immunopathogenicity of acanthamoeba spp. In the brain and lungs. Int J Mol Sci 2021; 22:1261. 33514026 10.3390/ijms22031261 PMC7865479

[2] Marciano-Cabral F, Cabral G. Acanthamoeba spp. As agents of disease in humans. Clin Microbiol Rev 2003; 16:273–307. 12692099 10.1128/CMR.16.2.273-307.2003 PMC153146

[3] Bunsuwansakul C, Mahboob T, Hounkong K, et al Acanthamoeba in Southeast Asia - overview and challenges. Korean J Parasitol 2019; 57:341–57. 31533401 10.3347/kjp.2019.57.4.341 PMC6753290

[4] Gupta N, Swathi Kiran PV, Khanna V, Khurana S, Varma M, Kumar TP. Motile trophozoites in granulomatous amoebic encephalitis. QJM Mon J Assoc Physicians 2025; .:hcaf048. 10.1093/qjmed/hcaf048 39945787

[5] Spottiswoode N, Haston JC, Hanners NW, et al Challenges and advances in the medical treatment of granulomatous amebic encephalitis. Ther Adv Infect Dis 2024; 11:20499361241228340. 38312848 10.1177/20499361241228340 PMC10838035

[6] Raju R, Khurana S, Mahadevan A, John DV. Central nervous system infections caused by pathogenic free-living amoebae: an Indian perspective. Trop Biomed 2022; 39:265–80. 35838101 10.47665/tb.39.2.017

[7] Kalra SK, Sharma P, Shyam K, Tejan N, Ghoshal U. Acanthamoeba and its pathogenic role in granulomatous amebic encephalitis. Exp Parasitol 2020; 208:107788. 31647916 10.1016/j.exppara.2019.107788

[8] Sarink MJ, van der Meijs NL, Denzer K, Koenderman L, Tielens AGM, van Hellemond JJ. Three encephalitis-causing amoebae and their distinct interactions with the host. Trends Parasitol 2022; 38:230–45. 34758928 10.1016/j.pt.2021.10.004

[9] Porritt K, Gomersall J, Lockwood C. JBI's systematic reviews: study selection and critical appraisal. Am J Nurs 2014; 114:47–52. 10.1097/01.NAJ.0000450430.97383.64 24869584

[10] Gelman BB, Popov V, Chaljub G, et al Neuropathological and ultrastructural features of amebic encephalitis caused by sappinia diploidea. J Neuropathol Exp Neurol 2003; 62:990–8. 14575235 10.1093/jnen/62.10.990

[11] Velayudhan G, Tom Thomas M, Kundoly VS, Joseph T, Aayiliath KA. Vermamoeba vermiformis causing primary amoebic meningoencephalitis - A diagnostic challenge. Trop Doct 2025; 55:128–30. 40095922 10.1177/00494755251327531

[12] Heilmann A, Rueda Z, Alexander D, Laupland KB, Keynan Y. Impact of climate change on amoeba and the bacteria they host. J Assoc Med Microbiol Infect Dis Can 2024;9:1–5. 10.3138/jammi-2023-09-08 PMC10984314 38567368

[13] Jung S, Schelper RL, Visvesvara GS, Chang HT. Balamuthia mandrillaris meningoencephalitis in an immunocompetent patient: an unusual clinical course and a favorable outcome. Arch Pathol Lab Med 2004; 128:466–8. 15043486 10.5858/2004-128-466-BMMIAI

[14] Bakardjiev A, Azimi PH, Ashouri N, et al Amebic encephalitis caused by Balamuthia mandrillaris: report of four cases. Pediatr Infect Dis J 2003; 22:447–52. 12792389 10.1097/01.inf.0000066540.18671.f8

[15] Schuster FL, Yagi S, Gavali S, et al Under the radar: Balamuthia amebic encephalitis. Clin Infect Dis Off Publ Infect Dis Soc Am 2009;48:879–87. 10.1086/597260 19236272

[16] Seijo Martínez M, González-Mediero G, Santiago P, et al Granulomatous amebic encephalitis in a patient with AIDS: isolation of acanthamoeba sp. Group II from brain tissue and successful treatment with sulfadiazine and fluconazole. J Clin Microbiol 2000; 38:3892–5. 11015431 10.1128/jcm.38.10.3892-3895.2000 PMC87504

[17] Damhorst GL, Watts A, Hernandez-Romieu A, et al Acanthamoeba castellanii encephalitis in a patient with AIDS: a case report and literature review. Lancet Infect Dis 2022; 22:e59–65. 34461057 10.1016/S1473-3099(20)30933-6 PMC10910629

[18] Voshtina E, Huang H, Raj R, Atallah E. Amebic encephalitis in a patient with chronic lymphocytic leukemia on ibrutinib therapy. Case Rep Hematol 2018; 2018:6514604. 30155323 10.1155/2018/6514604 PMC6092972

[19] Mungroo MR, Khan NA, Maciver S, Siddiqui R. Opportunistic free-living amoebal pathogens. Pathog Glob Health 2022; 116:70–84. 34602025 10.1080/20477724.2021.1985892 PMC8933017

[20] Vollmer ME, Glaser C. A balamuthia survivor. JMM Case Rep 2016; 3:e005031. 28348755 10.1099/jmmcr.0.005031 PMC5330223

[21] Wang L, Cheng W, Li B, et al Balamuthia mandrillaris infection in China: a retrospective report of 28 cases. Emerg Microbes Infect 2020; 9:2348–57. 33048025 10.1080/22221751.2020.1835447 PMC7599003

[22] Galarza M, Cuccia V, Sosa FP, Monges JA. Pediatric granulomatous cerebral amebiasis: a delayed diagnosis. Pediatr Neurol 2002; 26:153–6. 11897483 10.1016/s0887-8994(01)00360-5

[23] Deetz TR, Sawyer MH, Billman G, Schuster FL, Visvesvara GS. Successful treatment of Balamuthia amoebic encephalitis: presentation of 2 cases. Clin Infect Dis Off Publ Infect Dis Soc Am 2003;37:1304–12. 10.1086/379020 14583863

[24] Taallapalli AVR, Nashi S, Kulkarni GB, Alladi S, Chickabasaviah YT. Granulomatous amebic encephalitis presenting like a tumor-chasing a diagnostic conundrum. Ann Indian Acad Neurol 2021; 24:968–70. 35359565 10.4103/aian.AIAN_994_20 PMC8965954

[25] Benoit P, Wang S, Wang C, et al Brainstorm: a case of granulomatous encephalitis. J Assoc Med Microbiol Infect Dis Can J Off Assoc Pour Microbiol Medicale Infect Can 2024; 9:113–20. 10.3138/jammi-2023-0036 PMC12169439 40641815

[26] Baig AM . Proposals for amendments in the diagnosis and treatment of encephalitis caused by free-living amoebae. Infect Disord Drug Targets 2020; 20:115–21. 30961516 10.2174/1871526519666190405170601

[27] Visvesvara GS . Infections with free-living amebae. Handb Clin Neurol 2013; 114:153–68. 23829906 10.1016/B978-0-444-53490-3.00010-8

[28] Singh P, Kochhar R, Vashishta RK, et al Amebic meningoencephalitis: spectrum of imaging findings. AJNR Am J Neuroradiol 2006; 27:1217–21. 16775267 PMC8133936

[29] Mei J, Sheng F, Zhang C, Chen X. Imaging monitoring of Balamuthia granulomatous amoebic encephalitis. Clin Neurol Neurosurg 2025; 254:108917. 40300291 10.1016/j.clineuro.2025.108917

[30] Webster D, Umar I, Kolyvas G, et al Treatment of granulomatous amoebic encephalitis with voriconazole and miltefosine in an immunocompetent soldier. Am J Trop Med Hyg 2012; 87:715–8. 22869634 10.4269/ajtmh.2012.12-0100 PMC3516325

[31] Taravaud A, Loiseau PM, Pomel S. In vitro evaluation of antimicrobial agents on acanthamoeba sp. and evidence of a natural resilience to amphotericin B. Int J Parasitol Drugs Drug Resist 2017; 7:328–36. 28918001 10.1016/j.ijpddr.2017.09.002 PMC5604952

[32] Roelens M, Neehus AL, Rosain J, et al Cerebral amebiasis due to *Acanthamoeba* sp. In a patient with complete gp91 ^phox^ deficiency. J Hum Immun 2026; 2:e20250160. 41816252 10.70962/jhi.20250160 PMC12974230

