# Supplementary material for: Clinical Profile, Epidemiology, and Outcomes of Granulomatous Amebic Encephalitis: A Systematic Review
Source: Open Forum Infect Dis. 2026 May 12;13(5):ofag289. doi: 10.1093/ofid/ofag289 (PMC13182718; doi:10.1093/ofid/ofag289)
Supplement: ofag289_Supplementary_Data [file ofag289_supplementary_data.zip › Supplementary Table 1.docx]

**Supplementary Table 1: Baseline details of the authors and the countries from which individual cases of Granulomatous Acanthamoeba Encephalitis were reported** [1–142]

| Sn | Study ID | Case number | Country | Study type |
| --- | --- | --- | --- | --- |
| 1 | Haston 2025 [2] | 1 | USA | Case report |
| 2 | Alkhunaizi 2013 [3] | 1 | Saudi Arabia | Case report |
| 3 | Atamna 2025 [4] | 1 | Israel | Case report |
| 4 | Schafer 2015 [5] | 1 | USA | Case report |
| 5 | Hu 2022 [6] | 1 | China | Case report |
| 6 | Kim 2022 [7] | 1 | South Korea | Case report |
| 7 | Shehab 2018 [8] | 1 | USA | Case report |
| 8 | Sarica 2009 [9] | 1 | Turkey | Case report |
| 9 | Orozco 2011 [10] | 2 | USA | Case series |
| 10 | Monogue 2019 [11] | 1 | USA | Case report |
| 11 | Fu 2020 [12] | 1 | China | Case report |
| 12 | Fuke 2023 [13] | 1 | China | Case report |
| 13 | MartÃ­nCabello-VÃ­lchez 2024 [14] | 1 | Peru | Case report |
| 14 | Navarrete 2024 [15] | 1 | Chile | Case report |
| 15 | Alqam 2024 [16] | 1 | USA | Case report |
| 16 | Flores 2024 [17] | 1 | USA | Case report |
| 17 | Gullett 1979 [18] | 1 | USA | Case report |
| 18 | Lalitha 1985 [19] | 1 | India | Case report |
| 19 | Ofori-Kwakye 1986 [20] | 1 | USA | Case report |
| 20 | Harwood 1988 [21] | 1 | Australia | Case report |
| 21 | Feingold 1998 [22] | 1 | USA | Case report |
| 22 | Kidney 1998 [23] | 1 | USA | Case series |
| 23 | Katz 2000 [24] | 1 | USA | Case report |
| 24 | Deol 2000 [25] | 1 | Georgia | Case report |
| 25 | SeijoMartinez 2000 [26] | 1 | USA | Case report |
| 26 | Singhal 2001 [27] | 3 | India | Case series |
| 27 | Galarza 2002 [28] | 4 | USA | Case series |
| 28 | Bakardjiev 2003 [29] | 4 | USA | Case series |
| 29 | Velho 2003 [30] | 1 | India | Case report |
| 30 | Gelman 2003 [31] | 1 | USA | Case report |
| 31 | Deetz 2003 [32] | 2 | USA | Case series |
| 32 | Jung 2004 [33] | 1 | USA | Case report |
| 33 | Intalapaporn 2004 [34] | 1 | Thailand | Case report |
| 34 | Bloch 2005 [35] | 1 | USA | Case report |
| 35 | Yagi 2005 [36] | 5 | USA | Case series |
| 36 | Petry 2006 [37] | 1 | Germany | Case report |
| 37 | Tavares 2006 [38] | 1 | USA | Case report |
| 38 | OddÃ³B 2006 [39] | 1 | Chile | Case report |
| 39 | CuevasP 2006 [40] | 1 | Chile | Case report |
| 40 | Valverde 2006 [41] | 1 | Peru | Case report |
| 41 | Meersseman 2007 [42] | 1 | Austria | Case report |
| 42 | Silva-Vergara 2007 [43] | 1 | Brazil | Case report |
| 43 | Gupta 2008 [44] | 1 | India | Case report |
| 44 | Kaushal 2008 [45] | 1 | India | Case report |
| 45 | Ranjan 2009 [46] | 1 | India | Case report |
| 46 | Glaser C [47] | 10 | USA | Case series |
| 47 | Bodi 2008 [48] | 1 | UK | Case report |
| 48 | Kaul 2008 [49] | 1 | USA | Case report |
| 49 | Aichelburg 2008 [50] | 1 | Austria | Case report |
| 50 | Kansagra 2009 [51] | 1 | USA | Case report |
| 51 | Schuster 2009 [52] | 10 | USA | Case series |
| 52 | Sheng 2009 [53] | 1 | Taiwan | Case report |
| 53 | Lackner 2010 [54] | 1 | Austria | Case report |
| 54 | Saxena 2009 [55] | 1 | India | Case report |
| 55 | Cary 2010 [56] | 1 | USA | Case report |
| 56 | MartÃ­nez 2010 [57] | 1 | Peru | Case report |
| 57 | Schlessinger S [58] | 3 | USA | Case series |
| 58 | Doyle 2011 [59] | 1 | Australia | Case report |
| 59 | Maritschnegg 2011 [60] | 1 | Austria | Case report |
| 60 | Silva 2010 [61] | 1 | Brazil | Case report |
| 61 | Orozco 2011 [62] | 1 | USA | Case report |
| 62 | Yamasaki 2011 [63] | 1 | Japan | Case report |
| 63 | Stidd 2012 [64] | 1 | USA | Case report |
| 64 | Bando 2012 [65] | 1 | Japan | Case report |
| 65 | Webster 2012 [66] | 1 | Canada | Case report |
| 66 | Khurana 2012 [67] | 1 | India | Case report |
| 67 | Afshar 2013 [68] | 1 | USA | Case report |
| 68 | Krasaelap 2013 [69] | 1 | Thailand | Case report |
| 69 | Qvarnstrom 2013 [70] | 1 | USA | Case report |
| 70 | Kato 2013 [71] | 1 | Japan | Case report |
| 71 | Satlin 2013 [72] | 1 | USA | Case report |
| 72 | Lobo 2013 [73] | 1 | USA | Case report |
| 73 | Moriarty 2014 [74] | 1 | Australia | Case report |
| 74 | Chandra 2014 [75] | 1 | India | Case report |
| 75 | Zamora 2014 [76] | 1 | USA | Case report |
| 76 | Khanna 2014 [77] | 1 | India | Case report |
| 77 | Azzam 2015 [78] | 1 | Australia | Case report |
| 78 | Dowell 2015 [79] | 1 | USA | Case report |
| 79 | Salameh 2015 [80] | 1 | USA | Case report |
| 80 | Wilson 2015 [81] | 1 | USA | Case report |
| 81 | Roy 2015 [82] | 1 | USA | Case report |
| 82 | Thamtam 2016 [83] | 1 | India | Case report |
| 83 | Farnon 2016 [84] | 6 | USA | Case series |
| 84 | Gunawan 2016 [85] | 1 | Indonesia | Case report |
| 85 | Vollmer 2016 [86] | 1 | USA | Case report |
| 86 | ElSahly 2017 [87] | 1 | USA | Case report |
| 87 | Lehmer 2017 [88] | 1 | USA | Case report |
| 88 | Geith 2018 [89] | 1 | Germany | Case report |
| 89 | Voshtina 2018 [90] | 1 | USA | Case report |
| 90 | Piper 2018 [91] | 1 | USA | Case report |
| 91 | Harrison 2018 [92] | 1 | USA | Case report |
| 92 | SÃ¼tÃ§Ã¼ 2018 [93] | 1 | Turkey | Case report |
| 93 | Yohannan 2019 [94] | 1 | USA | Case report |
| 94 | Kum 2019 [95] | 1 | South Korea | Case report |
| 95 | Lau 2021 [96] | 1 | USA | Case report |
| 96 | Yang 2020 [97] | 1 | China | Case report |
| 97 | Cabello-VÃ­lchez 2020 [98] | 2 | Peru | Case series |
| 98 | Suzuki 2020 [99] | 1 | Japan | Case report |
| 99 | Crothers 2020 [100] | 1 | USA | Case report |
| 100 | Das 2020 [101] | 2 | India | Case series |
| 101 | Wu 2020 [102] | 1 | China | Case report |
| 102 | Keane 2020 [103] | 1 | Ireland | Case report |
| 103 | Kalyatanda 2020 [104] | 1 | USA | Case report |
| 104 | Suyo-Prieto 2021 [105] | 1 | Peru | Case report |
| 105 | Wang 2020 [106] | 14 | China | Case series |
| 106 | Yi 2021 [107] | 1 | China | Case report |
| 107 | Aparicio 2021 [108] | 1 | Mexico | Case report |
| 108 | Ã–calDemir 2021 [109] | 1 | Turkey | Case report |
| 109 | Damhorst 2022 [110] | 1 | USA | Case report |
| 110 | SolÃ­s-Castro 2021 [111] | 3 | Peru | Case series |
| 111 | Zhang 2022 [112] | 1 | China | Case report |
| 112 | Cuoco 2022 [113] | 1 | USA | Case report |
| 113 | Paudel 2021 [114] | 1 | USA | Case report |
| 114 | Chan 2022 [115] | 1 | Australia | Case report |
| 115 | Peng 2022 [116] | 1 | China | Case report |
| 116 | Xu 2022 [117] | 1 | China | Case report |
| 117 | Tao 2022 [118] | 1 | China | Case report |
| 118 | Tootla 2022 [119] | 1 | South Africa | Case report |
| 119 | Levinson 2022 [120] | 1 | USA | Case report |
| 120 | Spottiswoode 2023 [121] | 1 | USA | Case report |
| 121 | Chowdhury 2023 [122] | 1 | India | Case report |
| 122 | Fan 2023 [123] | 1 | China | Case report |
| 123 | Liu 2023 [124] | 1 | China | Case report |
| 124 | Yao 2023 [125] | 1 | China | Case report |
| 125 | Ono 2024 [126] | 1 | Japan | Case report |
| 126 | Haldar 2024 [127] | 3 | India | Case series |
| 127 | Qin 2024 [128] | 1 | China | Case report |
| 128 | Xu 2024 [129] | 1 | China | Case report |
| 129 | Li 2024 [130] | 1 | China | Case report |
| 130 | Qin 2024 [131] | 1 | China | Case report |
| 131 | Javed 2024 [132] | 1 | Pakistan | Case report |
| 132 | Carija 2025 [133] | 1 | Australia | Case report |
| 133 | Zheng 2024 [134] | 1 | China | Case report |
| 134 | Aboubechara 2025 [135] | 1 | USA | Case report |
| 135 | Liang 2025 [136] | 1 | China | Case report |
| 136 | Edminster 2025 [137] | 1 | USA | Case report |
| 137 | Velayudhan 2025 [138] | 2 | India | Case series |
| 138 | Liang 2025 [139] | 1 | China | Case report |
| 139 | Mei 2025 [140] | 1 | China | Case report |
| 140 | Pramanik 2025 [141] | 1 | India | Case report |
| 141 | Benoit 2024 [142] | 1 | Canada | Case report |
| 142 | Chowdhury 2025 [1] | 1 | India | Case report |

Bibliography:

1. Chowdhury S, Priyanka Y, Agarwal A, Garg D, Garg A, Jangir H, et al. Relapsing granulomatous amoebic encephalitis. Pract Neurol. 2025;pn-2025-004674. https://doi.org/10.1136/pn-2025-004674

2. Haston JC, Ali IK, Roy S, Roundtree A, Hofstetter J, Pierson S, et al. Notes from the Field: Fatal Acanthamoeba Encephalitis in a Patient Who Regularly Used Tap Water in an Electronic Nasal Irrigation Device and a Continuous Positive Airway Pressure Machine at Home - New Mexico, 2023. MMWR Morb Mortal Wkly Rep. 2025;74:179–80. https://doi.org/10.15585/mmwr.mm7410a4

3. Alkhunaizi AM, Dawamneh MF, Banda RW, Daabil RA, Al-Tawfiq JA, Akkad SA, et al. Acanthamoeba encephalitis in a patient with systemic lupus treated with rituximab. Diagn Microbiol Infect Dis. 2013;75:192–4. https://doi.org/10.1016/j.diagmicrobio.2012.11.003

4. Atamna A, Franken L, Grossman T, Rozenblatt S, Tobar A, Fichman-Horn S, et al. Acanthamoeba healyi (genotype T12) meningoencephalitis in an adult – first case in Israel. Diagn Microbiol Infect Dis. 2025;113:116988. https://doi.org/10.1016/j.diagmicrobio.2025.116988

5. Schafer KR, Shah N, Almira-Suarez MI, Reese JM, Hoke GM, Mandell JW, et al. Disseminated Balamuthia mandrillaris Infection. J Clin Microbiol. 2015;53:3072–6. https://doi.org/10.1128/JCM.01549-15

6. Hu J, Zhang Y, Yu Y, Yu H, Guo S, Shi D, et al. Encephalomyelitis Caused by Balamuthia mandrillaris in a Woman With Breast Cancer: A Case Report and Review of the Literature. Front Immunol. 2022;12:768065. https://doi.org/10.3389/fimmu.2021.768065

7. Kim JY, Yi M-H, Kim M, Yeom J-S, Yoo HD, Kim SM, et al. Diagnosis of Balamuthia mandrillaris Encephalitis by Thymine-Adenine Cloning Using Universal Eukaryotic Primers. Ann Lab Med. 2022;42:196–202. https://doi.org/10.3343/alm.2022.42.2.196

8. Shehab KW, Aboul-Nasr K, Elliott SP. Balamuthia mandrillaris Granulomatous Amebic Encephalitis With Renal Dissemination in a Previously Healthy Child: Case Report and Review of the Pediatric Literature. J Pediatr Infect Dis Soc. 2018;7:e163–8. https://doi.org/10.1093/jpids/pix089

9. Sarica FB, Tufan K, Cekinmez M, Erdoğan B, Altinörs MN. A rare but fatal case of granulomatous amebic encephalitis with brain abscess: the first case reported from Turkey. Turk Neurosurg. 2009;19:256–9.

10. Orozco L, Hanigan W, Khan M, Fratkin J, Lee M. Neurosurgical intervention in the diagnosis and treatment of Balamuthia mandrillaris encephalitis. J Neurosurg. 2011;115:636–40. https://doi.org/10.3171/2011.4.JNS102057

11. Monogue ML, Watson D, Alexander JS, Cavuoti D, Doyle LM, Wang MZ, et al. Minimal Cerebrospinal Fluid Concentration of Miltefosine despite Therapeutic Plasma Levels during the Treatment of Amebic Encephalitis. Antimicrob Agents Chemother. 2019;64:e01127-19. https://doi.org/10.1128/AAC.01127-19

12. Fu NX, Song J, Huang X, Lin GH. Granulomatous amoebic encephalitis presenting as a solitary mass lesion. Radiol Infect Dis. 2020;7:204–7. https://doi.org/10.1016/j.jrid.2020.09.001

13. Fuke T, Ogawa T, Shindo A, Araki M, Ishikawa M, Yagita K, et al. A Case of Granulomatous Amoebic Encephalitis diagnosed and intervened at an Early Stage. Jpn J Neurosurg. 2023;32:261–7. https://doi.org/10.7887/jcns.32.261

14. Martín Cabello-Vílchez A, Isabel Ruiz-Ruiz M. Molecular analysis unmasking a Balamuthia mandrillaris: Skin lesion and granulomatous amebic encephalitis by Acanthamoeba sp close to genotype T4 with fatal outcome. Clin Infect Pract. 2024;21:100246. https://doi.org/10.1016/j.clinpr.2023.100246

15. Navarrete J, Oyarce A, Oliva B, Lozano C, Amarales C, Cordero EM, et al. Encefalitis amebiana granulomatosa por Balamuthia mandrillaris en Chile confirmada con secuenciación de ADN. Rev Chil Infectol. 2024;41:176–83. https://doi.org/10.4067/s0716-10182024000100176

16. Alqam A, Jackson D, Griffin L. A RARE CASE OF GRANULOMATOUS AMEBIC ENCEPHALITIS IN A PATIENT WITH NEUROENDOCRINE TUMOR AND CUSHING’S SYNDROME. CHEST. Elsevier; 2024;166:A3020–1. https://doi.org/10.1016/j.chest.2024.06.1818

17. Flores MR, Montion MG, Usman AN, Ngene MA, Ruxmohan S. Granulomatous amebic encephalitis in a patient treated with chemotherapy: a case report and literature review. J Neurocritical Care. 2024;17:88–93. https://doi.org/10.18700/jnc.240032

18. Gullett J, Mills J, Hadley K, Podemski B, Pitts L, Gelber R. Disseminated granulomatous acanthamoeba infection presenting as an unusual skin lesion. Am J Med. 1979;67:891–6. https://doi.org/10.1016/0002-9343(79)90750-2

19. Lalitha MK, Anandi V, Srivastava A, Thomas K, Cherian AM, Chandi SM. Isolation of Acanthamoeba culbertsoni from a patient with meningitis. J Clin Microbiol. 1985;21:666–7. https://doi.org/10.1128/jcm.21.4.666-667.1985

20. Ofori-Kwakye SK, Sidebottom DG, Herbert J, Fischer EG, Visvesvara GS. Granulomatous brain tumor caused by Acanthamoeba. Case report. J Neurosurg. 1986;64:505–9. https://doi.org/10.3171/jns.1986.64.3.0505

21. Harwood CR, Rich GE, McAleer R, Cherian C. Isolation of *Acanthamoeba* from a cerebral abscess. Med J Aust. 1988;148:47–9. https://doi.org/10.5694/j.1326-5377.1988.tb104486.x

22. Feingold J, Abraham J, Bilgrami S, Ngo N, Visvesara G, Edwards R, et al. Acanthamoeba meningoencephalitis following autologous peripheral stem cell transplantation. Bone Marrow Transplant. 1998;22:297–300. https://doi.org/10.1038/sj.bmt.1701320

23. Kidney DD, Kim SH. CNS infections with free-living amebas: neuroimaging findings. AJR Am J Roentgenol. 1998;171:809–12. https://doi.org/10.2214/ajr.171.3.9725321

24. Katz JD, Ropper AH, Adelman L, Worthington M, Wade P. A case of Balamuthia mandrillaris meningoencephalitis. Arch Neurol. 2000;57:1210–2. https://doi.org/10.1001/archneur.57.8.1210

25. Deol I, Robledo L, Meza A, Visvesvara GS, Andrews RJ. Encephalitis due to a free-living amoeba (Balamuthia mandrillaris): Case report with literature review. Surg Neurol. 2000;53:611–6. https://doi.org/10.1016/S0090-3019(00)00232-9

26. Seijo Martinez M, Gonzalez-Mediero G, Santiago P, Rodriguez De Lope A, Diz J, Conde C, et al. Granulomatous amebic encephalitis in a patient with AIDS: isolation of acanthamoeba sp. Group II from brain tissue and successful treatment with sulfadiazine and fluconazole. J Clin Microbiol. 2000;38:3892–5. https://doi.org/10.1128/JCM.38.10.3892-3895.2000

27. Singhal T, Bajpai A, Kalra V, Kabra SK, Samantaray JC, Satpathy G, et al. Successful treatment of Acanthamoeba meningitis with combination oral antimicrobials. Pediatr Infect Dis J. 2001;20:623–7. https://doi.org/10.1097/00006454-200106000-00016

28. Galarza M, Cuccia V, Sosa FP, Monges JA. Pediatric granulomatous cerebral amebiasis: a delayed diagnosis. Pediatr Neurol. 2002;26:153–6. https://doi.org/10.1016/S0887-8994(01)00360-5

29. Bakardjiev A, Azimi PH, Ashouri N, Ascher DP, Janner D, Schuster FL, et al. Amebic encephalitis caused by Balamuthia mandrillaris: report of four cases. Pediatr Infect Dis J. 2003;22:447–52. https://doi.org/10.1097/01.inf.0000066540.18671.f8

30. Velho V, Sharma GK, Palande DA. Cerebrospinal acanthamebic granulomas. Case report. J Neurosurg. 2003;99:572–4. https://doi.org/10.3171/jns.2003.99.3.0572

31. Gelman BB, Popov V, Chaljub G, Nader R, Rauf SJ, Nauta HW, et al. Neuropathological and ultrastructural features of amebic encephalitis caused by Sappinia diploidea. J Neuropathol Exp Neurol. 2003;62:990–8. https://doi.org/10.1093/jnen/62.10.990

32. Deetz TR, Sawyer MH, Billman G, Schuster FL, Visvesvara GS. Successful treatment of Balamuthia amoebic encephalitis: presentation of 2 cases. Clin Infect Dis Off Publ Infect Dis Soc Am. 2003;37:1304–12. https://doi.org/10.1086/379020

33. Jung S, Schelper RL, Visvesvara GS, Chang HT. Balamuthia mandrillaris meningoencephalitis in an immunocompetent patient: an unusual clinical course and a favorable outcome. Arch Pathol Lab Med. 2004;128:466–8. https://doi.org/10.5858/2004-128-466-BMMIAI

34. Intalapaporn P, Suankratay C, Shuangshoti S, Phantumchinda K, Keelawat S, Wilde H. Balamuthia mandrillaris meningoencephalitis: the first case in southeast Asia. Am J Trop Med Hyg. 2004;70:666–9.

35. Bloch KC, Schuster FL. Inability to make a premortem diagnosis of Acanthamoeba species infection in a patient with fatal granulomatous amebic encephalitis. J Clin Microbiol. 2005;43:3003–6. https://doi.org/10.1128/JCM.43.6.3003-3006.2005

36. Yagi S, Booton GC, Visvesvara GS, Schuster FL. Detection of Balamuthia mitochondrial 16S rRNA gene DNA in clinical specimens by PCR. J Clin Microbiol. 2005;43:3192–7. https://doi.org/10.1128/JCM.43.7.3192-3197.2005

37. Petry F, Torzewski M, Bohl J, Wilhelm-Schwenkmezger T, Scheid P, Walochnik J, et al. Early diagnosis of Acanthamoeba infection during routine cytological examination of cerebrospinal fluid. J Clin Microbiol. 2006;44:1903–4. https://doi.org/10.1128/JCM.44.5.1903-1904.2006

38. Tavares M, Correia da Costa JM, Carpenter SS, Santos LA, Afonso C, Aguiar A, et al. Diagnosis of first case of Balamuthia amoebic encephalitis in Portugal by immunofluorescence and PCR. J Clin Microbiol. 2006;44:2660–3. https://doi.org/10.1128/JCM.00479-06

39. Oddó B D, Ciani A S, Vial C P. [Granulomatous amebic encephalitis caused by Balamuthia mandrillaris. First case diagnosed in Chile]. Rev Chil Infectologia Organo Of Soc Chil Infectologia. 2006;23:232–6.

40. Cuevas P M, Smoje P G, Jofré M L, Ledermann D W, Noemí H I, Berwart C F, et al. [Granulomatous amoebic meningoencephalitis by Balamuthia mandrillaris: case report and literature review]. Rev Chil Infectologia Organo Of Soc Chil Infectologia. 2006;23:237–42. https://doi.org/10.4067/s0716-10182006000300007

41. Valverde J, Arrese JE, Piérard GE. Granulomatous cutaneous centrofacial and meningocerebral amebiasis. Am J Clin Dermatol. 2006;7:267–9. https://doi.org/10.2165/00128071-200607040-00009

42. Meersseman W, Lagrou K, Sciot R, de Jonckheere J, Haberler C, Walochnik J, et al. Rapidly fatal Acanthamoeba encephalitis and treatment of cryoglobulinemia. Emerg Infect Dis. 2007;13:469–71. https://doi.org/10.3201/eid1303.061001

43. Silva-Vergara ML, Da Cunha Colombo ER, De Figueiredo Vissotto E, Silva ACAL, Chica JEL, Etchebehere RM, et al. Disseminated Balamuthia mandrillaris amoeba infection in an AIDS patient from Brazil. Am J Trop Med Hyg. 2007;77:1096–8.

44. Gupta D, Panda GS, Bakhshi S. Successful treatment of acanthamoeba meningoencephalitis during induction therapy of childhood acute lymphoblastic leukemia. Pediatr Blood Cancer. 2008;50:1292–3. https://doi.org/10.1002/pbc.21477

45. Kaushal V, Chhina DK, Kumar R, Pannu HS, Dhooria HPS, Chhina RS. Acanthamoeba encephalitis. Indian J Med Microbiol. 2008;26:182–4. https://doi.org/10.4103/0255-0857.40539

46. Ranjan R, Handa A, Choudhary A, Kumar S. Acanthamoeba infection in an interhemispheric ependymal cyst: a case report. Surg Neurol. 2009;72:185–9. https://doi.org/10.1016/j.surneu.2008.04.008

47. Centers for Disease Control and Prevention (CDC). Balamuthia amebic encephalitis--California, 1999-2007. MMWR Morb Mortal Wkly Rep. 2008;57:768–71.

48. Bodi I, Dutt N, Hampton T, Akbar N. Fatal granulomatous amoebic meningoencephalitis due to Balamuthia mandrillaris. Pathol - Res Pract. 2008;204:925–8. https://doi.org/10.1016/j.prp.2008.06.005

49. Kaul DR, Lowe L, Visvesvara GS, Farmen S, Khaled YA, Yanik GA. Acanthamoeba infection in a patient with chronic graft-versus-host disease occurring during treatment with voriconazole. Transpl Infect Dis Off J Transplant Soc. 2008;10:437–41. https://doi.org/10.1111/j.1399-3062.2008.00335.x

50. Aichelburg AC, Walochnik J, Assadian O, Prosch H, Steuer A, Perneczky G, et al. Successful treatment of disseminated Acanthamoeba sp. infection with miltefosine. Emerg Infect Dis. 2008;14:1743–6. https://doi.org/10.3201/eid1411.070854

51. Kansagra AP, Menon JP, Yarbrough CK, Urbaniak K, Waters JD, Borys E, et al. Balamuthia mandrillaris meningoencephalitis in an immunocompromised patient. Case report. J Neurosurg. 2009;111:301–5. https://doi.org/10.3171/2008.9.JNS08718

52. Schuster FL, Yagi S, Gavali S, Michelson D, Raghavan R, Blomquist I, et al. Under the radar: balamuthia amebic encephalitis. Clin Infect Dis Off Publ Infect Dis Soc Am. 2009;48:879–87. https://doi.org/10.1086/597260

53. Sheng W-H, Hung C-C, Huang H-H, Liang S-Y, Cheng Y-J, Ji D-D, et al. First case of granulomatous amebic encephalitis caused by Acanthamoeba castellanii in Taiwan. Am J Trop Med Hyg. 2009;81:277–9.

54. Lackner P, Beer R, Broessner G, Helbok R, Pfausler B, Brenneis C, et al. Acute granulomatous acanthamoeba encephalitis in an immunocompetent patient. Neurocrit Care. 2010;12:91–4. https://doi.org/10.1007/s12028-009-9291-z

55. Saxena A, Mittal S, Burman P, Garg P. Acanthameba meningitis with successful outcome. Indian J Pediatr. 2009;76:1063–4. https://doi.org/10.1007/s12098-009-0205-z

56. Cary LC, Maul E, Potter C, Wong P, Nelson PT, Given C, et al. Balamuthia mandrillaris meningoencephalitis: survival of a pediatric patient. Pediatrics. 2010;125:e699-703. https://doi.org/10.1542/peds.2009-1797

57. Martínez DY, Seas C, Bravo F, Legua P, Ramos C, Cabello AM, et al. Successful treatment of Balamuthia mandrillaris amoebic infection with extensive neurological and cutaneous involvement. Clin Infect Dis Off Publ Infect Dis Soc Am. 2010;51:e7-11. https://doi.org/10.1086/653609

58. Schlessinger S. Balamuthia mandrillaris Transmitted Through Organ Transplantation — Mississippi, 2009. Am J Transplant. Elsevier; 2011;11:173–6. https://doi.org/10.1111/j.1600-6143.2010.03395_1.x

59. Doyle JS, Campbell E, Fuller A, Spelman DW, Cameron R, Malham G, et al. Balamuthia mandrillaris brain abscess successfully treated with complete surgical excision and prolonged combination antimicrobial therapy. J Neurosurg. 2011;114:458–62. https://doi.org/10.3171/2010.10.JNS10677

60. Maritschnegg P, Sovinz P, Lackner H, Benesch M, Nebl A, Schwinger W, et al. Granulomatous amebic encephalitis in a child with acute lymphoblastic leukemia successfully treated with multimodal antimicrobial therapy and hyperbaric oxygen. J Clin Microbiol. 2011;49:446–8. https://doi.org/10.1128/JCM.01456-10

61. Silva RAE, Araújo S de A, Avellar IF de FE, Pittella JEH, Oliveira JT de, Christo PP. Granulomatous amoebic meningoencephalitis in an immunocompetent patient. Arch Neurol. 2010;67:1516–20. https://doi.org/10.1001/archneurol.2010.309

62. Orozco LD, Khan MA, Fratkin JD, Hanigan WC. Asymptomatic aneurysm of the cavernous and supraclinoid internal carotid artery in a patient with Balamuthia mandrillaris encephalitis. J Clin Neurosci Off J Neurosurg Soc Australas. 2011;18:1118–20. https://doi.org/10.1016/j.jocn.2010.11.033

63. Yamasaki K, Sugimoto T, Futami M, Moriyama T, Uehara H, Takeshima H, et al. Granulomatous amoebic encephalitis caused by Balamuthia mandrillaris. Neurol Med Chir (Tokyo). 2011;51:667–70. https://doi.org/10.2176/nmc.51.667

64. Stidd DA, Root B, Weinand ME, Anton R. Granulomatous amoebic encephalitis caused by Balamuthia mandrillaris in an immunocompetent girl. World Neurosurg. 2012;78:715.e7-12. https://doi.org/10.1016/j.wneu.2011.10.040

65. Bando Y, Takahashi T, Uehara H, Kagegi T, Nagahiro S, Izumi K. Autopsy case of amebic granulomatous meningoencephalitis caused by Balamuthia mandrillaris in Japan. Pathol Int. 2012;62:418–23. https://doi.org/10.1111/j.1440-1827.2012.02816.x

66. Webster D, Umar I, Kolyvas G, Bilbao J, Guiot M-C, Duplisea K, et al. Treatment of granulomatous amoebic encephalitis with voriconazole and miltefosine in an immunocompetent soldier. Am J Trop Med Hyg. 2012;87:715–8. https://doi.org/10.4269/ajtmh.2012.12-0100

67. Khurana S, Mewara A, Verma S, Totadri SK. Central nervous system infection with Acanthamoeba in a malnourished child. BMJ Case Rep. 2012;2012:bcr2012007449. https://doi.org/10.1136/bcr-2012-007449

68. Afshar K, Boydking A, Ganesh S, Herrington C, McFadden PM. Rapidly fatal disseminated acanthamoebiasis in a single lung transplant recipient. Ann Transplant. 2013;18:108–11. https://doi.org/10.12659/AOT.883846

69. Krasaelap A, Prechawit S, Chansaenroj J, Punyahotra P, Puthanakit T, Chomtho K, et al. Fatal Balamuthia amebic encephalitis in a healthy child: a case report with review of survival cases. Korean J Parasitol. 2013;51:335–41. https://doi.org/10.3347/kjp.2013.51.3.335

70. Qvarnstrom Y, Nerad TA, Visvesvara GS. Characterization of a new pathogenic Acanthamoeba Species, A. byersi n. sp., isolated from a human with fatal amoebic encephalitis. J Eukaryot Microbiol. 2013;60:626–33. https://doi.org/10.1111/jeu.12069

71. Kato H, Mitake S, Yuasa H, Hayashi S, Hara T, Matsukawa N. Successful treatment of granulomatous amoebic encephalitis with combination antimicrobial therapy. Intern Med Tokyo Jpn. 2013;52:1977–81. https://doi.org/10.2169/internalmedicine.52.0299

72. Satlin MJ, Graham JK, Visvesvara GS, Mena H, Marks KM, Saal SD, et al. Fulminant and fatal encephalitis caused by Acanthamoeba in a kidney transplant recipient: case report and literature review. Transpl Infect Dis Off J Transplant Soc. 2013;15:619–26. https://doi.org/10.1111/tid.12131

73. Lobo SA, Patil K, Jain S, Marks S, Visvesvara GS, Tenner M, et al. Diagnostic challenges in Balamuthia mandrillaris infections. Parasitol Res. 2013;112:4015–9. https://doi.org/10.1007/s00436-013-3592-z

74. Moriarty P, Burke C, McCrossin D, Campbell R, Cherian S, Shahab MS, et al. Balamuthia mandrillaris Encephalitis: Survival of a Child With Severe Meningoencephalitis and Review of the Literature. J Pediatr Infect Dis Soc. 2014;3:e4-9. https://doi.org/10.1093/jpids/pit033

75. Chandra SR, Adwani S, Mahadevan A. Acanthamoeba meningoencephalitis. Ann Indian Acad Neurol. 2014;17:108–12. https://doi.org/10.4103/0972-2327.128571

76. Zamora A, Henderson H, Swiatlo E. Acanthamoeba encephalitis: A Case Report and Review of Therapy. Surg Neurol Int. 2014;5:68. https://doi.org/10.4103/2152-7806.132239

77. Khanna V, Shastri B, Anusha G, Mukhopadhayay C, Khanna R. Acanthamoeba meningoencephalitis in immunocompetent: A case report and review of literature. Trop Parasitol. 2014;4:115–8. https://doi.org/10.4103/2229-5070.138540

78. Azzam R, Badenoch PR, Francis MJ, Fernandez C, Adamson PJ, Dendle C, et al. Acanthamoeba Encephalitis: Isolation of Genotype T1 in Mycobacterial Liquid Culture Medium. Diekema DJ, editor. J Clin Microbiol. 2015;53:735–9. https://doi.org/10.1128/JCM.02887-14

79. Dowell JD, Mukherjee S, Raghavan P, Rehm PK. AIDS Presenting as Granulomatous Amebic Encephalitis: PET and MR Imaging correlation. J Neuroimaging Off J Am Soc Neuroimaging. 2015;25:1047–9. https://doi.org/10.1111/jon.12212

80. Salameh A, Bello N, Becker J, Zangeneh T. Fatal Granulomatous Amoebic Encephalitis Caused by Acanthamoeba in a Patient With Kidney Transplant: A Case Report. Open Forum Infect Dis. 2015;2:ofv104. https://doi.org/10.1093/ofid/ofv104

81. Wilson MR, Shanbhag NM, Reid MJ, Singhal NS, Gelfand JM, Sample HA, et al. Diagnosing Balamuthia mandrillaris Encephalitis With Metagenomic Deep Sequencing. Ann Neurol. 2015;78:722–30. https://doi.org/10.1002/ana.24499

82. Roy SL, Atkins JT, Gennuso R, Kofos D, Sriram RR, Dorlo TPC, et al. Assessment of blood-brain barrier penetration of miltefosine used to treat a fatal case of granulomatous amebic encephalitis possibly caused by an unusual Balamuthia mandrillaris strain. Parasitol Res. 2015;114:4431–9. https://doi.org/10.1007/s00436-015-4684-8

83. Thamtam VK, Uppin MS, Pyal A, Kaul S, Rani JY, Sundaram C. Fatal granulomatous amoebic encephalitis caused by Acanthamoeba in a newly diagnosed patient with systemic lupus erythematosus. Neurol India. 2016;64:101–4. https://doi.org/10.4103/0028-3886.173662

84. Farnon EC, Kokko KE, Budge PJ, Mbaeyi C, Lutterloh EC, Qvarnstrom Y, et al. Transmission of Balamuthia mandrillaris by Organ Transplantation. Clin Infect Dis Off Publ Infect Dis Soc Am. 2016;63:878–88. https://doi.org/10.1093/cid/ciw422

85. Gunawan PI, Idarto A, Saharso D. Acanthamoeba Infection in a Drowning Child. Ethiop J Health Sci. 2016;26:289–92. https://doi.org/10.4314/ejhs.v26i3.12

86. Vollmer ME, Glaser C. A Balamuthia survivor. JMM Case Rep. 2016;3:e005031. https://doi.org/10.1099/jmmcr.0.005031

87. El Sahly H, Udayamurthy M, Parkerson G, Hasbun R. Survival of an AIDS patient after infection with Acanthamoeba sp. of the central nervous system. Infection. 2017;45:715–8. https://doi.org/10.1007/s15010-017-1037-9

88. Lehmer LM, Ulibarri GE, Ragsdale BD, Kunkle J. Cutaneous Balamuthia mandrillaris infection as a precursor to Balamuthia amoebic encephalitis (BAE) in a healthy 84-year-old Californian. Dermatol Online J. 2017;23:13030/qt8c8720qm.

89. Geith S, Walochnik J, Prantl F, Sack S, Eyer F. Lethal outcome of granulomatous acanthamoebic encephalitis in a man who was human immunodeficiency virus-positive: a case report. J Med Case Reports. 2018;12:201. https://doi.org/10.1186/s13256-018-1734-8

90. Voshtina E, Huang H, Raj R, Atallah E. Amebic Encephalitis in a Patient with Chronic Lymphocytic Leukemia on Ibrutinib Therapy. Case Rep Hematol. 2018;2018:6514604. https://doi.org/10.1155/2018/6514604

91. Piper KJ, Foster H, Susanto D, Maree CL, Thornton SD, Cobbs CS. Fatal Balamuthia mandrillaris brain infection associated with improper nasal lavage. Int J Infect Dis IJID Off Publ Int Soc Infect Dis. 2018;77:18–22. https://doi.org/10.1016/j.ijid.2018.09.013

92. Harrison WT, Lecky B, Hulette CM. Fatal Granulomatous Amebic Encephalitis in a Heart Transplant Patient: Clinical, Radiographic, and Autopsy Findings. J Neuropathol Exp Neurol. 2018;77:1001–4. https://doi.org/10.1093/jnen/nly089

93. Sütçü M, Aktürk H, Gülümser-Şişko S, Acar M, Erol OB, Somer A, et al. Granulomatous amebic encephalitis caused by Acanthamoeba in an immuncompetent child. Turk J Pediatr. 2018;60:340–3. https://doi.org/10.24953/turkjped.2018.03.019

94. Yohannan B, Feldman M. Fatal Balamuthia mandrillaris Encephalitis. Case Rep Infect Dis. 2019;2019:9315756. https://doi.org/10.1155/2019/9315756

95. Kum SJ, Lee HW, Jung HR, Choe M, Kim SP. Amoebic Encephalitis Caused by Balamuthia mandrillaris. J Pathol Transl Med. 2019;53:327–31. https://doi.org/10.4132/jptm.2019.05.14

96. Lau HL, De Lima Corvino DF, Guerra FM, Malik AM, Lichtenberger PN, Gultekin SH, et al. Granulomatous amoebic encephalitis caused by Acanthamoeba in a patient with AIDS: a challenging diagnosis. Acta Clin Belg. 2021;76:127–31. https://doi.org/10.1080/17843286.2019.1660023

97. Yang Y, Hu X, Min L, Dong X, Guan Y. Balamuthia mandrillaris-Related Primary Amoebic Encephalitis in China Diagnosed by Next Generation Sequencing and a Review of the Literature. Lab Med. 2020;51:e20–6. https://doi.org/10.1093/labmed/lmz079

98. Cabello-Vílchez AM, Chura-Araujo MA, Anicama Lima WE, Vela C, Asencio AY, García H, et al. Fatal granulomatous amoebic encephalitis due to free-living amoebae in two boys in two different hospitals in Lima, Perú. Neuropathol Off J Jpn Soc Neuropathol. 2020;40:180–4. https://doi.org/10.1111/neup.12617

99. Suzuki T, Okamoto K, Genkai N, Kakita A, Abe H. A homogeneously enhancing mass evolving into multiple hemorrhagic and necrotic lesions in amoebic encephalitis with necrotizing vasculitis. Clin Imaging. 2020;60:48–52. https://doi.org/10.1016/j.clinimag.2019.10.015

100. Crothers JW, Hsu L, Marty FM. Fulminant Acanthamoeba castellanii Encephalitis in an Ibrutinib-Treated Patient. Open Forum Infect Dis. 2020;7:ofaa025. https://doi.org/10.1093/ofid/ofaa025

101. Das S, Gunasekaran K, Ajjampur SSR, Abraham D, George T, Janeela MA, et al. Acanthamoeba encephalitis in immunocompetent hosts: A report of two cases. J Fam Med Prim Care. 2020;9:1240–3. https://doi.org/10.4103/jfmpc.jfmpc_1010_19

102. Wu X, Yan G, Han S, Ye Y, Cheng X, Gong H, et al. Diagnosing Balamuthia mandrillaris encephalitis via next-generation sequencing in a 13-year-old girl. Emerg Microbes Infect. 2020;9:1379–87. https://doi.org/10.1080/22221751.2020.1775130

103. Keane NA, Lane LM, Canniff E, Hare D, Doran S, Wallace E, et al. A Surviving Case of Acanthamoeba Granulomatous Amebic Encephalitis in a Hematopoietic Stem Cell Transplant Recipient. Am J Case Rep. 2020;21:e923219. https://doi.org/10.12659/AJCR.923219

104. Kalyatanda G, Rand K, Lindner MS, Hong DK, Sait Albayram M, Gregory J, et al. Rapid, Noninvasive Diagnosis of Balamuthia mandrillaris Encephalitis by a Plasma-Based Next-Generation Sequencing Test. Open Forum Infect Dis. 2020;7:ofaa189. https://doi.org/10.1093/ofid/ofaa189

105. Suyo-Prieto F, Núñez J, Guzmán K, Mostajo F, de Amat F, Ruiz M, et al. [First case report of the Balamuthia mandrillaris in the Camaná district of Arequipa, Peru]. Rev Argent Microbiol. 2021;53:129–34. https://doi.org/10.1016/j.ram.2020.05.002

106. Wang L, Cheng W, Li B, Jian Z, Qi X, Sun D, et al. Balamuthia mandrillaris infection in China: a retrospective report of 28 cases. Emerg Microbes Infect. 2020;9:2348–57. https://doi.org/10.1080/22221751.2020.1835447

107. Yi Z, Zhong J, Wu H, Li X, Chen Y, Chen H, et al. Balamuthia mandrillaris encephalitis in a child: case report and literature review. Diagn Microbiol Infect Dis. 2021;100:115180. https://doi.org/10.1016/j.diagmicrobio.2020.115180

108. Aparicio DV, Bejarano JIC, de Los Santos AM, Ramírez-Cortinas S, de la O Cavazos M. Case Report: Granulomatous Amebic Encephalitis due to Acanthamoeba spp. in an Immunocompetent Pediatric Patient. Am J Trop Med Hyg. 2021;105:167–70. https://doi.org/10.4269/ajtmh.21-0129

109. Öcal Demir S, Besli GE, Bucak A, Boral Ö, Baysal B. Successful Treatment of Acanthamoeba Meningoencephalitis in an Immunocompetent Child. J Trop Pediatr. 2021;67:fmaa102. https://doi.org/10.1093/tropej/fmaa102

110. Damhorst GL, Watts A, Hernandez-Romieu A, Mel N, Palmore M, Ali IKM, et al. Acanthamoeba castellanii encephalitis in a patient with AIDS: a case report and literature review. Lancet Infect Dis. 2022;22:e59–65. https://doi.org/10.1016/S1473-3099(20)30933-6

111. Solís-Castro ME. Lethal encephalitis due to free-living amoebae in three members of a family, in Tumbes, Peru. Rev Peru Med Exp Salud Publica. 2021;38:291–5. https://doi.org/10.17843/rpmesp.2021.382.6754

112. Zhang Z, Liang J, Wei R, Feng X, Wang L, Wang L, et al. Facial Balamuthia mandrillaris infection with neurological involvement in an immunocompetent child. Lancet Infect Dis. 2022;22:e93–100. https://doi.org/10.1016/S1473-3099(21)00334-0

113. Cuoco JA, Klein BJ, LeBel DP, Faulhaber J, Apfel LS, Witcher MR. Successful Treatment of a Balamuthia mandrillaris Cerebral Abscess in a Pediatric Patient With Complete Surgical Resection and Antimicrobial Therapy. Pediatr Infect Dis J. 2022;41:e54–7. https://doi.org/10.1097/INF.0000000000003418

114. Paudel AC, Patel N, Quang J, Casella C, Sigal A, Parajuli P, et al. Rapidly Progressive Granulomatous Amoebic Encephalitis in a Diabetic Individual. Cureus. 2021;13:e19336. https://doi.org/10.7759/cureus.19336

115. Chan A, Smith S, Tan E, Kuruvath S. Case Report: First Successful Treatment of Acanthamoeba Brain Abscess with Combination Surgical Excision and Miltefosine-Led Antimicrobial Therapy. Am J Trop Med Hyg. 2022;106:861–6. https://doi.org/10.4269/ajtmh.21-0166

116. Peng L, Zhou Q, Wu Y, Cao X, Lv Z, Su M, et al. A patient with granulomatous amoebic encephalitis caused by Balamuthia mandrillaris survived with two excisions and medication. BMC Infect Dis. 2022;22:54. https://doi.org/10.1186/s12879-021-07020-8

117. Xu C, Wu X, Tan M, Wang D, Wang S, Wu Y. Subacute Balamuthia mandrillaris encephalitis in an immunocompetent patient diagnosed by next-generation sequencing. J Int Med Res. 2022;50:3000605221093217. https://doi.org/10.1177/03000605221093217

118. Tao K, Wang T, Zhang L, Yang X-C, Zhai Z-F. Fatal Balamuthia mandrillaris infection with red plaques on the nasal dorsum as the first presentation. An Bras Dermatol. 2022;97:498–500. https://doi.org/10.1016/j.abd.2021.12.001

119. Tootla HD, Eley BS, Enslin JMN, Frean JA, Hlela C, Kilborn TN, et al. Balamuthia mandrillaris Granulomatous Amoebic Encephalitis: The First African Experience. J Pediatr Infect Dis Soc. 2022;11:578–81. https://doi.org/10.1093/jpids/piac096

120. Levinson S, Kumar KK, Wang H, Tayyar R, Dunning M, Toland A, et al. Balamuthia mandrillaris brain infection: a rare cause of a ring-enhancing central nervous system lesion. Illustrative case. J Neurosurg Case Lessons. 2022;3:CASE2268. https://doi.org/10.3171/CASE2268

121. Spottiswoode N, Pet D, Kim A, Gruenberg K, Shah M, Ramachandran A, et al. Successful Treatment of Balamuthia mandrillaris Granulomatous Amebic Encephalitis with Nitroxoline. Emerg Infect Dis. 2023;29:197–201. https://doi.org/10.3201/eid2901.221531

122. Chowdhury M, Prakash PR, Singh A, Jorwal P, Das S, Soneja M. Acanthamoeba meningoencephalitis causing secondary hemophagocytic lymphohistiocytosis in an immunocompetent patient: A first case report. J R Coll Physicians Edinb. 2023;53:23–6. https://doi.org/10.1177/14782715221149709

123. Fan X, Chen T, Yang H, Gao Y, Chen Y. Encephalomyelomeningitis Caused by Balamuthia mandrillaris: A Case Report and Literature Review. Infect Drug Resist. 2023;Volume 16:727–33. https://doi.org/10.2147/IDR.S400692

124. Liu J, Zhang W, Wu S, Zeng T, Luo F, Jiang Q, et al. A clinical case report of Balamuthia granulomatous amoebic encephalitis in a non-immunocompromised patient and literature review. BMC Infect Dis. 2023;23:245. https://doi.org/10.1186/s12879-023-08228-6

125. Yao S, Chen X, Qian L, Sun S, Zhao C, Bai Z, et al. Diagnosing Balamuthia mandrillaris amebic meningoencephalitis in a 64-year-old woman from the Southwest of China. Parasites Hosts Dis. 2023;61:183–93. https://doi.org/10.3347/PHD.23039

126. Ono Y, Higashida K, Yamanouchi K, Nomura S, Hanamatsu Y, Saigo C, et al. Balamuthia mandrillaris amoebic encephalitis mimicking tuberculous meningitis. Neuropathol Off J Jpn Soc Neuropathol. 2024;44:68–75. https://doi.org/10.1111/neup.12932

127. Haldar SN, Banerjee K, Modak D, Mondal A, Sharma C, Vasireddy T, et al. Case Report: A Series of Three Meningoencephalitis Cases Caused by Acanthamoeba spp. from Eastern India. Am J Trop Med Hyg. 2024;110:246–9. https://doi.org/10.4269/ajtmh.23-0396

128. Qin L, Xiang Y, Wu Z, Zhang H, Wu X, Chen Q. Metagenomic next-generation sequencing for diagnosis of fatal Balamuthia amoebic encephalitis. Infect Genet Evol J Mol Epidemiol Evol Genet Infect Dis. 2024;119:105570. https://doi.org/10.1016/j.meegid.2024.105570

129. Xu H, Wang D, Cui K, Wan R, Chi Q, Wu T. 18F-FDG PET/CT findings in fatal Balamuthia Mandrillaris encephalitis in brain stem: A case report. Radiol Case Rep. 2024;19:1851–4. https://doi.org/10.1016/j.radcr.2024.02.021

130. Li Z, Li W, Li Y, Ma F, Li G. A case report of Balamuthia mandrillaris encephalitis. Heliyon. 2024;10:e26905. https://doi.org/10.1016/j.heliyon.2024.e26905

131. Qin B, Jia L, Chen J, Liu W. Balamuthia Amebic Encephalitis Cured and Discharged. J Craniofac Surg. 2024; https://doi.org/10.1097/SCS.0000000000010123

132. Javed Z, Hussain MM, Ghanchi N, Gilani A, Enam SA. Non-granulomatous meningoencephalitis with Balamuthia mandrillaris mimicking a tumor: First confirmed case from Pakistan. Surg Neurol Int. 2024;15:238. https://doi.org/10.25259/SNI_181_2024

133. Carija SC, Levy A, Weaire‐Buchanan G, Lee T, Woodward R, Gazeley J, et al. Amoebic encephalitis within Australia. Intern Med J. 2025;55:143–7. https://doi.org/10.1111/imj.16523

134. Zheng Z, Chen F, Qin L, Lu A, Xu H, Zhao M, et al. Application of ventriculoscopy in granulomatous amoebic encephalitis: a case report in China and literature review. Front Med. 2024;11:1431225. https://doi.org/10.3389/fmed.2024.1431225

135. Aboubechara JP, Kantamneni T, Pasao K. Balamuthia Mandrillaris Central Nervous System Vasculitis in an Immunocompetent Child: Case Report. J Child Neurol. 2025;40:366–70. https://doi.org/10.1177/08830738241307058

136. Liang Y, Wang W. A Balamuthia amoebic encephalitis survivor in China, and literature review. Diagn Microbiol Infect Dis. 2025;111:116698. https://doi.org/10.1016/j.diagmicrobio.2025.116698

137. Edminster SY, Rebbe RW, Khatchadourian C, Hurth KM, Mathew AJ, Huss-Bawab J, et al. The role of plasma metagenomic sequencing in identification of Balamuthia mandrillaris encephalitis. Acta Neuropathol Commun. 2025;13:60. https://doi.org/10.1186/s40478-025-01963-8

138. Velayudhan G, Tom Thomas M, Kundoly V S, Joseph T, Aayiliath K A. Vermamoeba vermiformis causing primary amoebic meningoencephalitis - A diagnostic challenge. Trop Doct. 2025;55:128–30. https://doi.org/10.1177/00494755251327531

139. Liang Y, Liu Y, Chen Z, Sun J, Zhang X, Wang Y. Balamuthia amoebic encephalitis directly causing intracranial infection: A case report. Radiol Case Rep. 2025;20:2820–4. https://doi.org/10.1016/j.radcr.2025.02.035

140. Mei J, Sheng F, Zhang C, Chen X. Imaging monitoring of Balamuthia granulomatous amoebic encephalitis. Clin Neurol Neurosurg. 2025;254:108917. https://doi.org/10.1016/j.clineuro.2025.108917

141. Pramanik S, Dasgupta S, Samui S, Chandra A, Kattady FJ, Makhal M. Acute meningoencephalitis with subdural empyema associated with Acanthamoeba in an immunocompetent individual. J R Coll Physicians Edinb. 2025;55:184–7. https://doi.org/10.1177/14782715251342120

142. Benoit P, Wang S, Wang C, Chakravarti A, Villalba JA, Ali IKM, et al. Brainstorm: A Case of Granulomatous Encephalitis. J Assoc Med Microbiol Infect Dis Can J Off Assoc Pour Microbiol Medicale Infect Can. 2024;9:113–20. https://doi.org/10.3138/jammi-2023-0036
