# Supplementary material for: Clinical Profile, Epidemiology, and Outcomes of Granulomatous Amebic Encephalitis: A Systematic Review
Source: Open Forum Infect Dis. 2026 May 12;13(5):ofag289. doi: 10.1093/ofid/ofag289 (PMC13182718; doi:10.1093/ofid/ofag289)
Supplement: ofag289_Supplementary_Data [file ofag289_supplementary_data.zip › Supplementary Table 2.docx]

Supplementary Table 2: JBI Critical Appraisal of case reports included in the systematic reviews

| Sn | Author | Demography | History | Presentation | Diagnosis | Treatment | Follow-up | Adverse event |
| --- | --- | --- | --- | --- | --- | --- | --- | --- |
| 1 | Haston J | Yes | No | Yes | Yes | No | Yes | NA |
| 2 | Alkhunaizi A | Yes | No | Yes | Yes | Yes | Yes | NA |
| 3 | Atamna A | Yes | No | Yes | Yes | Yes | Yes | NA |
| 4 | Schafer K | Yes | No | Yes | Yes | Yes | Yes | NA |
| 5 | Hu J | Yes | Yes | Yes | Yes | Yes | Yes | NA |
| 6 | Kim J | Yes | No | Yes | Yes | No | Yes | NA |
| 7 | Shehab K | Yes | Yes | Yes | Yes | Yes | Yes | NA |
| 8 | Sarica N | Yes | Yes | Yes | No | Yes | Yes | NA |
| 9 | Orozco L | Yes | Yes | Yes | Yes | No | Yes | NA |
| 10 | Orozco L | Yes | No | Yes | Yes | Yes | Yes | NA |
| 11 | Monogue M | Yes | No | Yes | Yes | No | No | NA |
| 12 | Fu X | Yes | Yes | Yes | No | Yes | Yes | NA |
| 13 | Fuke T | Yes | No | Yes | Yes | Yes | Yes | NA |
| 14 | Vilchez M | Yes | Yes | Yes | Yes | Yes | Yes | NA |
| 15 | Navarrete J | Yes | Yes | Yes | Yes | No | Yes | NA |
| 16 | Alqam A | Yes | Yes | Yes | No | No | Yes | NA |
| 17 | Flores M | Yes | Yes | Yes | Yes | No | No | NA |
| 18 | Gullett J | Yes | Yes | Yes | Yes | Yes | Yes | NA |
| 19 | Lalitha M | Yes | No | Yes | Yes | Yes | Yes | NA |
| 20 | Ofori-Kwakye S | Yes | Yes | Yes | Yes | Yes | No | NA |
| 21 | Harwood C | Yes | Yes | Yes | Yes | Yes | Yes | NA |
| 22 | Feingold J | Yes | No | No | Yes | No | Yes | NA |
| 23 | Kidney D | Yes | Yes | Yes | Yes | No | Yes | NA |
| 24 | Katz J | Yes | Yes | Yes | Yes | No | Yes | NA |
| 25 | Deol I | Yes | Yes | Yes | Yes | No | Yes | NA |
| 26 | Martinez M | Yes | Yes | Yes | Yes | Yes | Yes | NA |
| 27 | Singhal T | Yes | Yes | Yes | Yes | Yes | Yes | NA |
| 28 | Singhal T | Yes | Yes | Yes | Yes | Yes | Yes | NA |
| 29 | Singhal T | Yes | Yes | Yes | Yes | Yes | Yes | NA |
| 30 | Galarza M | Yes | No | Yes | Yes | Yes | Yes | NA |
| 31 | Galarza M | Yes | No | Yes | Yes | Yes | Yes | NA |
| 32 | Galarza M | Yes | Yes | Yes | Yes | Yes | Yes | NA |
| 33 | Galarza M | Yes | No | Yes | Yes | Yes | Yes | NA |
| 34 | Bakardjiev A | Yes | Yes | Yes | Yes | No | Yes | NA |
| 35 | Bakardjiev A | Yes | Yes | Yes | Yes | Yes | Yes | NA |
| 36 | Bakardjiev A | Yes | Yes | Yes | Yes | Yes | Yes | NA |
| 37 | Bakardjiev A | Yes | Yes | Yes | Yes | Yes | Yes | NA |
| 38 | Velho V | Yes | Yes | Yes | Yes | No | Yes | NA |
| 39 | Gelman B | Yes | Yes | Yes | Yes | Yes | No | NA |
| 40 | Deetz T | Yes | Yes | Yes | Yes | Yes | Yes | NA |
| 41 | Deetz T | Yes | No | Yes | Yes | Yes | Yes | NA |
| 42 | Jung S | Yes | Yes | Yes | Yes | Yes | Yes | NA |
| 43 | Intalapaporn P | Yes | Yes | Yes | Yes | No | Yes | NA |
| 44 | Bloch K | Yes | Yes | Yes | Yes | No | Yes | NA |
| 45 | Yagi S | Yes | No | No | Yes | No | No | NA |
| 46 | Yagi S | Yes | No | No | Yes | No | No | NA |
| 47 | Yagi S | Yes | No | No | Yes | No | No | NA |
| 48 | Yagi S | Yes | No | No | Yes | No | No | NA |
| 49 | Yagi S | Yes | No | No | Yes | No | No | NA |
| 50 | Petry F | Yes | No | Yes | Yes | Yes | Yes | NA |
| 51 | Tavares M | Yes | Yes | Yes | Yes | Yes | Yes | NA |
| 52 | Oddo D | Yes | No | Yes | Yes | Yes | Yes | NA |
| 53 | Cuevas M | Yes | Yes | Yes | Yes | No | Yes | NA |
| 54 | Valverde J | Yes | No | No | Yes | No | Yes | NA |
| 55 | Meersseman W | Yes | No | Yes | Yes | No | Yes | NA |
| 56 | Silva-Vergara M | Yes | Yes | Yes | Yes | No | Yes | NA |
| 57 | Gupta D | Yes | No | Yes | Yes | Yes | Yes | NA |
| 58 | Kaushal V | Yes | Yes | Yes | Yes | Yes | Yes | NA |
| 59 | Ranjan R | Yes | Yes | Yes | Yes | Yes | Yes | NA |
| 60 | Glaser C | Yes | No | Yes | Yes | No | Yes | NA |
| 61 | Glaser C | Yes | No | Yes | Yes | No | Yes | NA |
| 62 | Glaser C | Yes | No | Yes | Yes | No | Yes | NA |
| 63 | Glaser C | Yes | No | Yes | Yes | No | Yes | NA |
| 64 | Glaser C | Yes | No | Yes | Yes | No | Yes | NA |
| 65 | Glaser C | Yes | No | Yes | Yes | No | Yes | NA |
| 66 | Glaser C | Yes | No | Yes | Yes | No | Yes | NA |
| 67 | Glaser C | Yes | No | Yes | Yes | No | Yes | NA |
| 68 | Glaser C | Yes | No | Yes | Yes | No | Yes | NA |
| 69 | Glaser C | Yes | No | Yes | Yes | No | Yes | NA |
| 70 | Bodi I | Yes | No | Yes | Yes | Yes | Yes | NA |
| 71 | Kaul R | Yes | No | Yes | Yes | Yes | Yes | NA |
| 72 | Alexander C | Yes | No | Yes | Yes | Yes | Yes | NA |
| 73 | Kansagra A | Yes | No | Yes | Yes | Yes | Yes | NA |
| 74 | Schuster F | Yes | Yes | Yes | Yes | No | Yes | NA |
| 75 | Schuster F | Yes | Yes | Yes | Yes | No | Yes | NA |
| 76 | Schuster F | Yes | No | Yes | Yes | No | Yes | NA |
| 77 | Schuster F | Yes | Yes | Yes | Yes | No | Yes | NA |
| 78 | Schuster F | Yes | Yes | Yes | Yes | No | Yes | NA |
| 79 | Schuster F | Yes | Yes | Yes | Yes | No | Yes | NA |
| 80 | Schuster F | Yes | No | Yes | Yes | No | Yes | NA |
| 81 | Schuster F | Yes | Yes | Yes | Yes | No | Yes | NA |
| 82 | Schuster F | Yes | Yes | Yes | Yes | No | Yes | NA |
| 83 | Schuster F | Yes | No | Yes | Yes | No | Yes | NA |
| 84 | Sheng W | Yes | Yes | Yes | Yes | Yes | Yes | NA |
| 85 | Lackner P | Yes | Yes | Yes | Yes | Yes | Yes | NA |
| 86 | Saxena A | Yes | Yes | Yes | Yes | Yes | Yes | NA |
| 87 | Cary L | Yes | No | Yes | Yes | Yes | Yes | NA |
| 88 | Martinez D | Yes | Yes | Yes | Yes | Yes | Yes | NA |
| 89 | Schlessinger S | Yes | No | Yes | Yes | No | Yes | NA |
| 90 | Schlessinger S | Yes | No | Yes | Yes | Yes | Yes | NA |
| 91 | Schlessinger S | Yes | No | Yes | Yes | Yes | Yes | NA |
| 92 | Doyle J | Yes | Yes | Yes | Yes | Yes | Yes | NA |
| 93 | Maritschnegg P | Yes | No | Yes | Yes | Yes | Yes | NA |
| 94 | Silva R | Yes | Yes | Yes | Yes | No | Yes | NA |
| 95 | Orozco L | Yes | Yes | Yes | Yes | Yes | Yes | NA |
| 96 | Yamasaki K | Yes | Yes | Yes | Yes | No | Yes | NA |
| 97 | Stidd D | Yes | Yes | Yes | Yes | No | Yes | NA |
| 98 | Bando Y | Yes | No | Yes | Yes | No | Yes | NA |
| 99 | Webster D | Yes | No | Yes | Yes | Yes | Yes | NA |
| 100 | Khurana S | Yes | Yes | Yes | Yes | Yes | Yes | NA |
| 101 | Afshar K | Yes | No | Yes | Yes | No | Yes | NA |
| 102 | Krasaelap A | Yes | Yes | Yes | Yes | Yes | Yes | NA |
| 103 | Qvarnstrom Y | Yes | Yes | Yes | Yes | No | Yes | NA |
| 104 | Kato H | Yes | Yes | Yes | Yes | Yes | Yes | NA |
| 105 | Satlin M | Yes | Yes | Yes | Yes | No | Yes | NA |
| 106 | Lobo S | Yes | Yes | Yes | Yes | No | Yes | NA |
| 107 | Moriarty P | Yes | Yes | Yes | Yes | Yes | Yes | NA |
| 108 | Chandra R | Yes | Yes | Yes | Yes | No | Yes | NA |
| 109 | Zamora A | Yes | Yes | Yes | Yes | Yes | Yes | NA |
| 110 | Khanna V | Yes | Yes | Yes | Yes | Yes | Yes | NA |
| 111 | Azzam R | Yes | Yes | Yes | Yes | Yes | Yes | NA |
| 112 | Dowell J | Yes | Yes | Yes | Yes | Yes | Yes | NA |
| 113 | Salameh A | Yes | Yes | Yes | Yes | Yes | Yes | NA |
| 114 | Wilson M | Yes | No | Yes | Yes | No | Yes | NA |
| 115 | Roy S | Yes | Yes | Yes | Yes | Yes | Yes | NA |
| 116 | Thamtam V | Yes | Yes | Yes | Yes | No | Yes | NA |
| 117 | Farnon E | Yes | No | Yes | Yes | No | Yes | NA |
| 118 | Farnon E | Yes | No | Yes | Yes | Yes | Yes | NA |
| 119 | Farnon E | Yes | No | Yes | Yes | Yes | Yes | NA |
| 120 | Farnon E | Yes | Yes | Yes | Yes | No | Yes | NA |
| 121 | Farnon E | Yes | No | Yes | Yes | Yes | Yes | NA |
| 122 | Farnon E | Yes | Yes | Yes | Yes | Yes | Yes | NA |
| 123 | Gunawan P | Yes | No | Yes | Yes | Yes | Yes | NA |
| 124 | Vollmer M | Yes | Yes | Yes | Yes | Yes | Yes | NA |
| 125 | Sahly H | Yes | No | Yes | Yes | Yes | Yes | NA |
| 126 | Lehmer L | Yes | Yes | Yes | Yes | Yes | Yes | NA |
| 127 | Geith S | Yes | Yes | Yes | Yes | No | Yes | NA |
| 128 | Voshtina E | Yes | No | Yes | Yes | No | Yes | NA |
| 129 | Piper K | Yes | No | Yes | Yes | Yes | Yes | NA |
| 130 | Harrison W | Yes | Yes | Yes | Yes | No | Yes | NA |
| 131 | Sutcu M | Yes | No | Yes | Yes | Yes | Yes | NA |
| 132 | Yohannan B | Yes | Yes | Yes | Yes | Yes | Yes | NA |
| 133 | Kum S | Yes | No | Yes | Yes | Yes | Yes | NA |
| 134 | Lau H | Yes | Yes | Yes | Yes | No | Yes | NA |
| 135 | Yang M | Yes | Yes | Yes | Yes | No | Yes | NA |
| 136 | Cabello-Vilchez A | Yes | No | Yes | No | No | Yes | NA |
| 137 | Cabello-Vilchez A | Yes | No | Yes | No | No | Yes | NA |
| 138 | Suzuki T | Yes | No | Yes | Yes | No | Yes | NA |
| 139 | Crothers J | Yes | Yes | Yes | Yes | Yes | Yes | NA |
| 140 | Das S | Yes | Yes | Yes | Yes | Yes | Yes | NA |
| 141 | Das S | Yes | Yes | Yes | Yes | Yes | Yes | NA |
| 142 | Wu X | Yes | Yes | Yes | Yes | Yes | Yes | NA |
| 143 | Keane N | Yes | Yes | Yes | Yes | Yes | Yes | NA |
| 144 | Kalyatanda G | Yes | Yes | Yes | Yes | Yes | Yes | NA |
| 145 | Suyo-Prieto F | Yes | Yes | Yes | Yes | Yes | Yes | NA |
| 146 | Wang L | Yes | No | Yes | Yes | No | Yes | NA |
| 147 | Wang L | Yes | No | Yes | Yes | No | Yes | NA |
| 148 | Wang L | Yes | No | Yes | Yes | No | Yes | NA |
| 149 | Wang L | Yes | No | Yes | Yes | No | Yes | NA |
| 150 | Wang L | Yes | No | Yes | Yes | No | Yes | NA |
| 151 | Wang L | Yes | No | Yes | Yes | No | Yes | NA |
| 152 | Wang L | Yes | No | Yes | Yes | No | Yes | NA |
| 153 | Wang L | Yes | No | Yes | Yes | No | Yes | NA |
| 154 | Wang L | Yes | No | Yes | Yes | No | Yes | NA |
| 155 | Wang L | Yes | No | Yes | Yes | No | Yes | NA |
| 156 | Wang L | Yes | No | Yes | Yes | No | Yes | NA |
| 157 | Wang L | Yes | Yes | Yes | Yes | No | Yes | NA |
| 158 | Wang L | Yes | No | Yes | Yes | No | Yes | NA |
| 159 | Wang L | Yes | No | Yes | Yes | No | Yes | NA |
| 160 | Yi Z | Yes | Yes | Yes | Yes | No | Yes | NA |
| 161 | Aparicio D | Yes | Yes | Yes | Yes | No | Yes | NA |
| 162 | OcalDemir S | Yes | No | Yes | Yes | Yes | Yes | NA |
| 163 | Damhorst G | Yes | Yes | Yes | Yes | Yes | Yes | NA |
| 164 | Castro M | Yes | No | Yes | Yes | No | Yes | NA |
| 165 | Castro M | Yes | Yes | Yes | Yes | No | Yes | NA |
| 166 | Castro M | Yes | Yes | Yes | Yes | No | Yes | NA |
| 167 | Zhnag Z | Yes | Yes | Yes | Yes | No | Yes | NA |
| 168 | Cuoco J | Yes | Yes | Yes | Yes | Yes | Yes | NA |
| 169 | Paudel A | Yes | Yes | Yes | Yes | Yes | Yes | NA |
| 170 | Chan A | Yes | No | Yes | Yes | Yes | Yes | NA |
| 171 | Peng L | Yes | No | Yes | Yes | Yes | Yes | NA |
| 172 | Xu C | Yes | No | Yes | Yes | Yes | Yes | NA |
| 173 | Tao K | Yes | No | Yes | Yes | No | Yes | NA |
| 174 | Tootla H | Yes | No | Yes | Yes | Yes | Yes | NA |
| 175 | Levinson S | Yes | Yes | Yes | Yes | Yes | Yes | NA |
| 176 | Spottiswoode N | Yes | No | Yes | Yes | Yes | Yes | NA |
| 177 | Chowdhury M | Yes | Yes | Yes | Yes | Yes | Yes | NA |
| 178 | Fan X | Yes | No | Yes | Yes | Yes | Yes | NA |
| 179 | Liu J | Yes | Yes | Yes | Yes | Yes | Yes | NA |
| 180 | Yao S | Yes | Yes | Yes | Yes | No | Yes | NA |
| 181 | Ono Y | Yes | Yes | Yes | Yes | No | Yes | NA |
| 182 | Haldar S | Yes | Yes | Yes | Yes | Yes | Yes | NA |
| 183 | Haldar S | Yes | Yes | Yes | Yes | Yes | Yes | NA |
| 184 | Haldar S | Yes | Yes | Yes | Yes | Yes | Yes | NA |
| 185 | Qin L | Yes | Yes | Yes | Yes | No | Yes | NA |
| 186 | Xu H | Yes | Yes | Yes | Yes | No | No | NA |
| 187 | Li Z | Yes | No | Yes | Yes | No | Yes | NA |
| 188 | Qin B | Yes | Yes | Yes | Yes | No | Yes | NA |
| 189 | Javed Z | Yes | Yes | Yes | Yes | No | Yes | NA |
| 190 | Carija S | Yes | Yes | Yes | Yes | No | Yes | NA |
| 191 | Zheng Z | Yes | Yes | Yes | Yes | Yes | Yes | NA |
| 192 | Aboubechara John Paul | Yes | Yes | Yes | Yes | Yes | Yes | NA |
| 193 | Liang Y | Yes | No | Yes | Yes | Yes | Yes | NA |
| 194 | Edminster S | Yes | Yes | Yes | Yes | No | Yes | NA |
| 195 | Velayudhan G | Yes | No | Yes | Yes | Yes | Yes | NA |
| 196 | Velayudhan G | Yes | No | Yes | Yes | No | Yes | NA |
| 197 | Liang Y | Yes | No | Yes | Yes | No | Yes | NA |
| 198 | Mei J | Yes | No | Yes | Yes | Yes | Yes | NA |
| 199 | Pramanik S | Yes | Yes | Yes | Yes | Yes | Yes | NA |
| 200 | Benoit P | Yes | Yes | Yes | Yes | Yes | Yes | NA |
| 201 | Chowdhury S | Yes | Yes | Yes | Yes | Yes | Yes | NA |
